# Building consensus on the core clinical competencies and outcomes related to effective child and adolescent mental health services (CAMHS) crisis team provision: findings from a Delphi study

**DOI:** 10.1136/bmjment-2026-302719

**Published:** 2026-08-11

**Authors:** Clau Nader, Lucy Tindall, Kapil Sayal, Josephine Holland, Catherine Elizabeth Hewitt, Paul A Tiffin

**Affiliations:** 1 Department of Health Sciences, University of York, York, England, UK; 2 Institute of Mental Health, School of Medicine, University of Nottingham, Nottingham, England, UK; 3 The Hull York Medical School, University of York, York, England, UK

**Keywords:** Adolescent, Mental Health Services

## Abstract

**Background:**

Young peoples’ mental health is worsening, increasing demands on Child and Adolescent Mental Health Services (CAMHS) crisis teams. However, evidence is lacking regarding the effectiveness of such services. To pave the way for service developments and robust evaluation, it is crucial to establish a consensus on the core competencies for CAMHS crisis clinicians and the most desirable outcomes from their input.

**Objective:**

To elicit and establish consensus among experts, including those with relevant lived experience, regarding the most important staff competencies and outcomes in this service context.

**Methods:**

A three-round Delphi study was conducted with young people (aged 18–25 years with relevant lived experience of CAMHS crisis team input), parents/carers and mental health clinicians/academics. The study aimed to establish consensus (≥70% agreement) on which core staff competencies and outcomes were perceived as ‘essential’/‘very important’ in relation to CAMHS crisis services.

**Findings:**

Of 69 initial participants, 43 (62%, mainly young people) completed all three rounds. A broad consensus was reached, with over 90% of proposed items meeting the consensus threshold. The strongest agreement was found for competencies in the *therapeutic alliance* domain, particularly *cultural competency* (93%) and *validation* (91%). For the *case formulation* domain, a *holistic mental state examination* (91%) was highly prioritised. Key outcomes with high consensus included *improved communication with support networks* (91%), *better understanding of the young person* (88%), *feeling validated* (88%) and *reduced involuntary inpatient admissions* (88%). The findings were integrated into a Theory of Change-based logic model illustrating how these skills and knowledge could lead to the desired outcomes.

**Conclusions:**

Our results demonstrate a clear stakeholder consensus prioritising relational, validating and personalised approaches to assessment and intervention over an emphasis on administrative or diagnostic processes in CAMHS crisis care. The findings also highlight a desire to move from a perception of a paternalistic risk management style to collaborative, community-based support.

**Clinical implications:**

These findings underscore the need for crisis team clinicians to be trained and supported to deliver care that is relationship-based, trauma-informed and culturally responsive. These should potentially translate into better outcomes for young people, their families and the crisis team workforce itself.

**WHAT IS ALREADY KNOWN ON THIS TOPIC**

**WHAT THIS STUDY ADDS**

- This study establishes a strong expert consensus for the core staff competencies and outcomes perceived as most important specifically in relation to CAMHS crisis teams.

**HOW THIS STUDY MIGHT AFFECT RESEARCH, PRACTICE OR POLICY**

- These findings demonstrate that future CAMHS crisis team workforce support and service development should prioritise relational and culturally responsive staff skills alongside collaborative safety planning and consent-based family involvement.
- Future evaluative research protocols should include avoidance of involuntary hospital admissions as a key outcome.

## Introduction

 Globally, there has been a marked increase in mental health difficulties among young people, with rising levels of psychological distress, self-harm and suicidality. For example, recent UK data indicate that approximately 17% of young people aged 17–19 experience mental health difficulties likely to meet diagnostic thresholds.^1^ These trends have translated into growing demand for urgent mental health support, with substantial increases in crisis presentations to services. In the UK, referrals to Child and Adolescent Mental Health Services (CAMHS) crisis teams increased sharply in recent years.^2^

During periods of acute distress, young people may present with significant risks that require urgent assessment. Where professionals judge that community-based support is insufficient to ensure safety, inpatient admission may be considered. Such admissions are often geographically distant from family and support networks, or occur in developmentally inappropriate adult settings.^3^ Although inpatient care can be beneficial, it also carries recognised iatrogenic risks for young people with mental health difficulties.^4 5^ Consequently, there is a strong policy and clinical imperative to intervene effectively at the point of crisis in the community, both to reduce distress and risk and to prevent avoidable hospital admissions.

Mental health crisis services were developed with these goals in mind. Crisis resolution and home treatment teams for adults have been implemented internationally since the early 2000s, with mixed evidence regarding their effectiveness in reducing hospital admissions.^6 7^ More recently, similar models have been extended to children and young people in the UK. National policy committed to ensuring that all young people in England have access to 24/7 CAMHS crisis teams.^8^ Despite substantial investment, however, there remains limited evidence regarding whether CAMHS crisis teams improve outcomes for young people and families. Key uncertainties include whether such services reduce distress and self-harm, facilitate engagement with appropriate interventions or prevent inpatient admission.

There is some evidence relating to the impact of intensive community care (ICC) approaches generally, for those young people who may otherwise be considered for mental health inpatient admission. Experts agree that ICC teams are characterised as rapidly responsive services providing treatment primarily outside of hospital in community settings (eg, school or home). They tend to offer frequent contacts (at least twice weekly) and out-of-hours support, with clinicians carrying small caseloads (ie, <10 patients).^9^ One recent pilot study randomised 12-year-olds to 17-year-olds experiencing psychiatric emergencies (n=36) to ICC services (which included crisis and intensive home treatment (IHT) functions) or treatment as usual. Thirty-six adolescents were randomised during the internal pilot phase. The low recruitment rate precluded progression to a fully powered trial. However, there were some positive trends in favour of the ICC group, with improved service satisfaction, clinical symptoms, functioning and a shorter time to return to education, employment or training.^10^ Similarly, a systematic review and meta-analysis concluded that ICC approaches generally reduce utilisation of inpatient services and improve functioning and some symptoms in young people.^9^ Nevertheless, this review did not include any specific crisis teams. Indeed, studies of ICC services have been unable to isolate the specific effect of the responsive, crisis element of such teams.

Alongside questions of effectiveness, workforce sustainability within CAMHS represents a significant challenge. High staff turnover has been identified as a barrier to delivering consistent, high-quality care.^11^ Crisis and acute mental health settings may be particularly vulnerable, with evidence that emotionally intensive work is associated with burnout and reduced staff retention. Emotional labour—the requirement for clinicians to regulate their emotional responses while supporting distressed young people—has been highlighted as a key contributor to moral distress and workforce attrition. Strengthening training, supervision and skills development may therefore be critical both for improving care quality and for supporting staff well-being and retention.

The Strengthening Crisis Team Provision for Young People via Technology-enhanced Assessment and Training (SCriPT) project was developed to address these challenges. Its overarching aim was to inform the development of a digital training prototype to support CAMHS crisis team clinicians through supervision and skills development. The prototype can be viewed at https://claunader.bubbleapps.io/version-test and the associated manual at https://osf.io/q2gve/files/vtj6z. As a precursor to intervention development, a Delphi consensus study was conducted to identify agreement on the core competencies required for effective crisis work with young people. In addition, the study sought to establish consensus on outcomes that are most meaningful to young people, families and clinicians, thereby informing future service evaluation. For the purposes of this project, CAMHS Crisis Services and Teams were defined according to the description given in the NHS long-term plan and elaborated on in NHS England service specifications for ‘Urgent and Emergency Mental Health Care for Children and Young People’.^12 13^ Such services are characterised by:

1. Operating 24/7 and as a single point of access.
2. Conducting prompt assessments that can take place across a number of settings (eg, home, accident and emergency (A&E)).
3. Delivering brief responses and interventions, usually constituting several sessions over, at most, several weeks.

We included such teams irrespective of whether they had other intensive, community-focused functionality or services linked to them, such as ‘assertive outreach’ or IHT services.

The Delphi method is well established in health research as a structured approach to generating consensus where empirical evidence is limited.^14 15^ Consistent with WHO guidance emphasising the central role of lived experience in shaping mental health systems,^16^ this study incorporated diverse perspectives, including those of young people and families. Integrating lived experience expertise helps to democratise knowledge production and mitigate traditional power imbalances in research.^17^

Accordingly, the aims of this Delphi study were to:

1. Achieve consensus on outcomes of CAMHS crisis team involvement that are most meaningful to young people, families and clinicians.
2. Identify the core therapeutic competencies required for mental health professionals working in crisis settings for young people.

## Methods

### Participants

Three participant groups were included: (1) young adults aged 18–25 years with lived experience of youth mental health crisis support (eg, CAMHS crisis services); (2) parents or carers of young people with such experience; and (3) clinicians with current or previous experience of providing crisis care to young people, alongside academics specialising in youth mental health research. Young adults aged under 18 were not recruited due to feasibility, safeguarding and ethical constraints within the project timeframe. The upper age limit was selected to ensure that participants had recent experience of crisis services as currently delivered.

Participants were recruited through a variety of routes, including professional, academic and informal networks. This included word of mouth among academic and clinical colleagues, charity organisations, health and care enterprises, community centres and patient involvement groups and networks. Involvement@York and the Child Oriented Mental Health Innovation Collaborative (COMIC), for example, helped circulate an invitation with their online directories to facilitate recruitment and widen participation. The invitation consisted of brief written, accessible information on the study, as well as an infographic that summarised the participation process. This enabled potential participants to understand the purpose and nature of the Delphi exercise and contact the research team to ask any questions about how their data would be used before deciding whether to take part via informed-consent.

### Delphi process

A three-stage Delphi exercise was conducted using Qualtrics survey software between July and November 2024. Participants were assigned unique six-digit participant identifiers to enable response linkage across rounds while maintaining anonymity.

An initial model of clinical competencies and outcomes was presented in round 1 (see online supplemental table S1). Competency domains were derived from a review of evidence on interventions for self-harm and suicidality in young people,^18^ resulting in five domains: family and carer involvement, therapeutic alliance, case formulation, psychoeducation and lethal means restriction. Proposed outcomes were generated from a literature review of youth crisis services and integrated with findings from prior SCriPT focus groups conducted with multiple Public and Patient Involvement and Engagement (PPIE) networks.^19^

In round 1, participants selected competencies and outcomes they considered important for crisis team practice and evaluation, with the option to endorse all items within a domain and to suggest additional elements via free-text responses. Demographic data were also collected. Following data cleaning and anonymisation using Microsoft Excel, free-text responses were imported into NVivo V.12 (Lumivero, 2024) and analysed using reflexive thematic analysis.^20^ Initially, two members of the research team familiarised themselves with the free-text data (CN, PAT). One member (CN) produced initial codes with NVivo by extracting key words and/or phrases that could link to the proposed competencies and outcomes. The extracted codes were then organised into recurring or emerging themes. On various occasions themes overlapped across competency areas. For example, ‘Cultural competency' was mentioned repeatedly for *Therapeutic Alliance*, for *Family and Carer Involvement* and for *Case Formulation*. Codes and themes were then reviewed by the second coder (PAT) against the original text. Any disagreements were resolved via discussion. The second coder (PAT) then took the lead on remapping these themes onto the competency domains of the original framework. The other coder (CN) then reviewed this mapping and any disagreements were resolved via discussion. Thus, a mixed inductive–deductive approach was used; the initial codes and themes were derived inductively, according to the data. After this, a deductive approach was used to map these themes onto the original skills/knowledge framework. In addition, following peer review as part of the publication process, the final themes were slightly modified by removing the quality of clinical supervision as a code, as it is an external factor.

In round 2, participants rated each competency and outcome using a 5-point Likert scale (1=irrelevant to 5=essential). In round 3, participants were shown their previous ratings alongside group consensus scores from round 2 (mode or mean where two modes existed) and invited to confirm or amend their responses.

Finally, the elements identified as important with good consensus were integrated into a Theory of Change informed logic model.^21^ This postulated how the desirable knowledge and skills related to both intermediate outputs and activities, and, ultimately the most valued outcomes.

### Analysis

**Figure 1:**
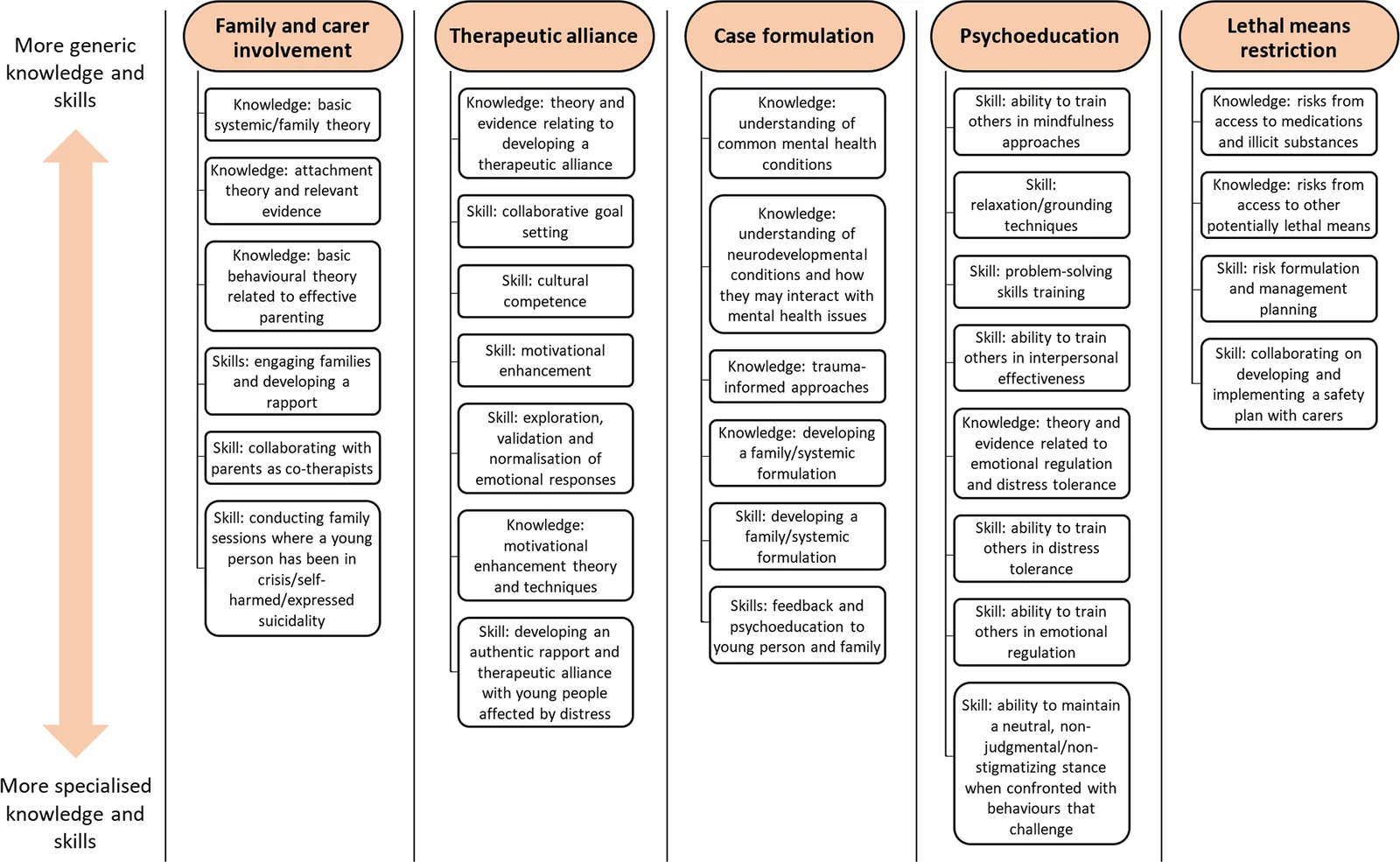
Set of core skills and knowledge domains that served as the foundation for the suggested list of competencies in the first round of the survey, based on Meza et al.^18^

Following completion of round 3, responses were categorised according to predefined consensus criteria: *consensus in* (≥70% ratings of 4–5 and <15% ratings of 1–2), *consensus out* (≥70% ratings of 1–2 and <15% ratings of 4–5) or *no consensus*.^22^ Items reaching consensus out were excluded, while remaining items were reviewed by the research team. Descriptive statistics (counts and percentages) were calculated. NVivo V.12 (Lumivero, 2024) was used to support thematic analysis of round 1 text responses, and quantitative analyses were conducted using Microsoft Excel, IBM SPSS Statistics (V.29) and Stata MP V.19.5. The latter was used to formally test for demographic differences between the participants who completed each stage of the Delphi exercise (χ^2^ for categorical variables and Mann-Whitney U tests for age). Result graphs were produced with Microsoft Excel using the magma colour scheme to enable accessibility for potential colourblind readers (figure 1).^23^

## Results

### Panel participant characteristics

In total, 69 participants took part in round 1 of the Delphi exercise, of whom 46 (66.7%) remained for round 2 and 43 (62%) for round 3. There was a range of participant types, age ranges, genders and ethnicities. The highest proportion of participants across each round of the survey were young people (see table 1). There were some statistically significant differences in the demographics of those completing the different stages. Young people and carers were more likely to complete stages 2 and 3 compared with clinicians and researchers (p=0.001 and p<0.001, respectively). Those completing stage 3 (compared with stage 1 only) were, on average, younger (p=0.02). No statistically significant differences in participant self-identified gender or ethnicity were observed across the stages.

**Table 1:** Participant demographics for each round of the Delphi survey

Characteristics	Round 1 (n=69)	Round 2 (n=46)	Round 3 (n=43)
Participant type			
Young person	32 (46.4%)	26 (56.5%)	26 (60.5%)
Parent/carer	18 (26.1%)	10 (21.8%)	10 (23.2%)
Clinician/practitioner	19 (27.5%)	9 (19.6%)	6 (14%)
Academic/researcher	7 (10.1%)	1 (2.2%)	1 (2.3%)
Age range			
18–30	35 (50.7%)	28 (60.9%)	28 (65.1%)
31–40	11 (15.9%)	3 (6.5%)	3 (7.0%)
41–50	11 (15.9%)	8 (17.4%)	6 (14.0%)
51 and over	12 (17.4%)	7 (15.2%)	6 (14.0%)
Gender			
Women	43 (60.9%)	28 (60.9%)	25 (58.1%)
Men	25 (36.2%)	17 (37.0%)	17 (39.5%)
Non-binary	2 (2.9%)	1 (2.2%)	1 (2.3%)
Ethnicity			
White	33 (47.8%)	19 (41.3%)	18 (41.9%)
Black	13 (18.8)	10 (21.7%)	9 (20.9%)
South Asian	13 (18.8%)	9 (19.6%)	8 (18.6%)
East Asian	2 (2.9%)	2 (4.3%)	2 (4.7%)
Arab or Middle Eastern	4 (5.8%)	4 (8.7%)	4 (9.3%)
Latin American	2 (2.9%)	1 (2.2%)	1 (2.3%)
Mixed ethnicity	1 (1.4%)	1 (2.2%)	1 (2.3%)
Prefer not to say	1 (1.4%)	0 (0.0%)	0 (0.0%)

### Round 1 results

Most participants endorsed the vast majority of the initial elements presented, indicating that they should be included in the remainder of the Delphi survey. The results of round 1 are shown in online supplemental table S2. In this regard the elements with the highest proportion of participants indicating that they should be included were *helping young people and families identify possible risks* (65/69, 94.2%); *working together with young people and their parents/carers to create a safety plan* (64/69, 92.8%); *exploration, validation and normalisation of emotional responses* (59/69, 85.5%); and *being knowledgeable about the existing risks and consequences of accessing potential harmful objects or substances* (59/69, 85.5%). In contrast, elements with the lowest proportion of endorsements were from the *Outcomes* domain; reduced chances of having to go to an A&E hospital department (37/69, 53.6%); reduced chances of going into a mental health inpatient unit, as a voluntary patient (39/69, 56.5%) or as an involuntary (‘detained’) patient (38/69, 55.1%).

The free text responses are shown in online supplemental table S3. The preliminary codes generated via the thematic analysis are also listed in section 2 of the online supplemental material. These relate to the suggestions for additional elements, highlighting potential gaps in the original key competencies model. The themes, elicited via the thematic analysis, were mapped, where possible, onto the existing competency domains originally proposed. These are depicted in table 2, along with brief, illustrative quotes from participants. For example, within the domain of *Case Formulation*, participants emphasised the need to situate any information and feedback within the appropriate cultural and personal context. For the *Family and Carer Involvement* domain, participants stressed the necessity of respecting the young person’s wishes. A new theme (subdomain) that emerged was taking into account cultural (including intersectional) factors when involving families in the assessment or care of a young person. In terms of the *Psychoeducation* domain, participants mentioned the possibility of using community-based interventions and spaces and that networks and societal influences should also be considered. For the *Therapeutic Alliance* domain suggestions included that personalised approaches, which account for cultural context, should be used. Moreover, new subdomains relating to developmentally sensitive approaches, staff supervision/support and self-reflection were introduced. In terms of *Lethal Means Restriction,* some participants emphasised long-term risk considerations and the role of contextual factors, including a family’s capacity to manage risk. For the *Outcomes* section, a novel element suggested was ‘*awareness of community-based spaces*’; that is, making young people and carers aware of such resources and how to access them.

**Table 2:** Additional elements suggested by participants during stage 1 of the Delphi exercise and how they may map on to the original domains presented

Domain	Initial elements	New elements suggested	Participant quotes
Case formulation	Being knowledgeable on common mental health difficulties	Holistic mental state examination	‘*Equally important to have a thorough and dynamic formulation*…’ (Practitioner)
		Role of digital environment	‘*Technology-enabled distress*, eg, *online bullying*…’ (Practitioner)
		Forensic risk assessment	‘*…access to means inc. firearms; technology assisted bullying(…) suicide websites*…’ (Practitioner)
		Up-to-date history file	‘*If …already on the CAMHS waiting list ensure that their file is up to date…’* (Researcher)
	Understanding neurodevelopmental conditions	Therapy interactions with neurodevelopmental conditions	‘*Understanding how neurodevelopmental conditions may interact with types of therapy*’ (Young Person)
	Understanding and responding to the impact of trauma	Trauma-informed care	‘*Context and its impact on the presenting crisis*’ (Researcher)
		Systemic trauma awareness	‘*Consider how the young person is being systemically oppressed*’ (Practitioner)
	Family/systemic formulation	Intrafamilial context understanding	‘*Understanding the psychopathology and how…families make sense of them*’ (Researcher)
		Experience mapping	‘*Which social care services have been utilised by the family (non-statutory as well as statutory)*’ (Practitioner)
	Providing information and feedback to young people and families	Personalised support	‘…*to understand better the individual’s situation and symptoms rather than being very generic and somewhat cliched*’ (Parent)
		Culture-informed assessment	‘*Cultural norms including traveller risks…Understanding a person’s background and environment*…’ (Practitioner)
Family and carer involvement	Knowledge of systemic/family theory; attachment theory	Intrafamilial context understanding	‘*…to be up-to-date with safeguarding training including parental mental health (PAMIC)…’* (Practitioner)
	Capacity to engage families and build rapport	Enabling proactive family engagement	*‘Involve families in different ways depending on the needs and wants of the young person…’* (Practitioner)
	Working jointly with parents/caregivers as co-therapists	Enabling consent-based family engagement	*‘Not all young people may want family involved…should first discuss with CYP…’* (Researcher)
	Hosting effective family meetings (distress, crisis, suicidality)	Informing patient’s consent for carer involvement	*‘They also should know that CYP can consent to interventions without parental consent even if under 16*’ (Researcher)
		Trauma-informed safeguarding	*‘The knowledge about when to not involve families if it would be unsafe*…’ (Young Person)
		Handling confidentiality	‘*Often the young person may not wish to have their situation involved with their parents and working out a way to bridge this gap if possible*’ (Young Person)
		Offering psychoeducation for carers	*‘Teaching parents to make self-soothe boxes for their young person’* (Practitioner)
	*(New/Emerging Theme*)	Intersectional culture-informed support approaches	‘*If it would ‘out’ someone to an unsupportive family’* (Young Person)
Psychoeducation	Teaching mindfulness; relaxation and grounding techniques	Intersectional self-care strategies	*‘Have these conversations in relation to aspects of identity…’* (Practitioner)
		Distraction techniques	*‘Leave the house and provide a helpful distraction*’ (Young Person)
	Helping young people manage emotions/healthy coping strategies	Compassion and acceptance-based self-regulation	‘*A strong knowledge of what is healthy or within the normal range of human behaviour*…’ (Practitioner-Researcher)
		Distress coping strategies	*‘Elements of DBT, [dialectical behaviour therapy] such as distress tolerance skills and emotional regulation skills’* (Young Person)
		Coping with uncertainty	*‘Sometimes things don't go to plan, uncertainty is a normal human experience’* (Practitioner-Researcher)
	Empowering young people to understand roots of behaviours	Societal hierarchies and oppressive systems	*‘Understanding the role [of] white supremacy, capitalism, individualism have on young people within society’* (Practitioner)
	Teaching positive communication and social-interaction skills	Effective communication skills with carers	*‘Ensuring parents do not dominate goal setting…Parents should also be taught …skills so that they can support the young person’* (Practitioner)
	Teaching young people and families where/how to seek help	How to ask for help	*‘Peer support avenues and how to help families access other forms of support, including self-help’* (Researcher)
		Community-based interventions and spaces	*‘It should be considered if support could be offered in settings other than just the home’* (Young Person)
		Resilience-building through support networks	*‘Empower young people by teaching them the therapeutic and healing qualities in nurturing community…’* (Practitioner)
		Self-awareness for relapse prevention	*‘Understanding their own personal relapse signature…recognising maintaining factors’* (Practitioner-Researcher)
Therapeutic alliance	Understanding how to build trust and rapport; normalisation of emotions	Person-centred individualised approach	*‘Learn to not use generic distress tolerance skills when they are known to not be useful…to the patient’* (Young Person)
		Empathy and compassion	*‘Often crisis teams suggest having a bath or a cup of tea and this is seen as not ’caring’ or not validating…’* (Young Person)
		Active listening	*‘What young people say is most important along with not having their feelings dismissed or downplayed’* (Researcher)
		Procuring a safe space	*‘All young people should have the choice…to have a separate mental health worker to the worker who is working with their caregivers’* (Practitioner)
	Collaborative goal-setting with young people and families	Collective or anti-oppressive goal setting	*‘Important that goal setting is not imposed’* (Practitioner)
	Cultural competence; cultural humility	Understanding of patients’ perception on diagnosis	*‘Understanding the psychopathology and how CYP/families make sense of them’* (Researcher)
	Motivational enhancement techniques	Youth understanding	*‘Trauma, including polyvagal theory and sensory integration responses to managing distress’* (Practitioner)
	*(New/Emerging Theme*)	Developmental stage-based approaches	*‘Developmental perspective - how to engage different ages’* (Practitioner)
	*(New/Emerging Theme*)	Self-reflective practice	*‘Open mindedness…good cognitive and emotional empathy skills’* (Young Person)
Lethal means restriction	Awareness of effects/dangers of misusing substances	Knowledge on common self-harm and suicide means	*‘Increase knowledge around risks that are not talked about as much such as the use of aerosols for self-harm or suicide’* (Practitioner)
	Understanding risks of access to harmful objects	Knowledge of contextual factors	*‘Ensuring that all contexts are considered to mitigate risk…’* (Practitioner)
		Understanding of the young person	*‘Understanding what the young person is going through and offering another type of escape than dangerous ones’* (Young Person)
	Collaborative creation of safety plans	Trauma-informed safeguarding approach	*‘Who is their trusted adult and not just default to parents’* (Researcher)
		Impact assessment of systemic barriers and enablers	*‘Cultural barriers and enablers/racism…’* (Parent) *‘…including developmental/systemic trauma for the parents’* (Practitioner)
		Hybrid modality of support	*‘Seeing the young person face to face not just on a crisis line call’* (Researcher)
		Understanding of existing safe spaces	*‘Checking basic needs are being met, that the young person has frequent optional access to a safe community…’* (Practitioner)
		Long-term considerations beyond crisis	*‘Not always useful in the long term to lock away or hide potential risks in the home…’* (Young Person)
		Behavioural risks implications for family	*‘…what the limits are to people’s ability to cope with risky behaviour’* (Researcher)
Outcomes	Receiving a mental health diagnosis and its implications	Ability to make informed-decisions	*‘An accurate diagnosis for young people who will benefit from this…to make informed decisions around this as much as possible’* (Practitioner)
		Better understanding of the young person	*‘Clearer formulation and understanding’* (Practitioner)
		Awareness of clinical diagnoses and social perceptions	*‘Give information about which diagnoses have negative stigma… and which diagnoses are more likely to lead to positive outcomes’* (Practitioner)
	Improved distress tolerance	Defined effective self-regulation strategies	*‘self-soothing strategies…regulating skills…’* (Researcher)
		Achievable plans for coping and adapting	*‘Improved plans for coping and adaptability…ability of family/carer to cope with this and future crises’* (Practitioner-Researcher)
	Reduced likelihood of further crises/suicidality	Reduced risks of suicide/self-harm	*‘Reduce risk of suicide or self-harm and…ensure they are referred [to CAMHS]*’ (Researcher)
	Reduced likelihood of hospital admission/A&E attendance	Assertive referral and access to long-term care	*‘Access to longer-term care…’* (Researcher)
	Improved family relationships	Improved support networks	*‘Increased connection with family or support’* (Practitioner)
		Improved communication with support networks	*‘Effective crisis intervention can help with overall engagement with mental health services’* (Practitioner)
	*(New/Emerging Theme*)	Awareness of community-based spaces	*‘Ensure the CYP and their family/carer are signposted to these sources of support’* (Practitioner)

A&E, accident and emergency; CAMHS, Child and Adolescent Mental Health Services; DBT, dialectical behaviour therapy.

### Round 2 results

In the second round, all elements presented in the first survey round were included, alongside the newly suggested elements. The full results of round 2, in terms of consensus, are presented in online supplemental table S4 and online supplemental figure S1. Almost all elements were viewed as *essential* or *very important*, with consensus (ie, ≥70% of the participants selecting these options). The elements with the highest level of consensus were effective *communication with carers* (93.5%), *psychoeducating carers* (91.3%) and *reduced risks of suicide/self-harm* (91.3%). In contrast, those elements with the lowest levels of consensus regarding their importance were *informed patient consent for carer involvement* (63%), *access to means for self-harm or suicide* (65.2% as part of the *Case Formulation* domain), mindfulness techniques (56.5%) and *impact assessment of systemic barriers and enablers* (56.5% as part of the *Lethal Means Safety* domain).

### Round 3 results

**Figure 2:**
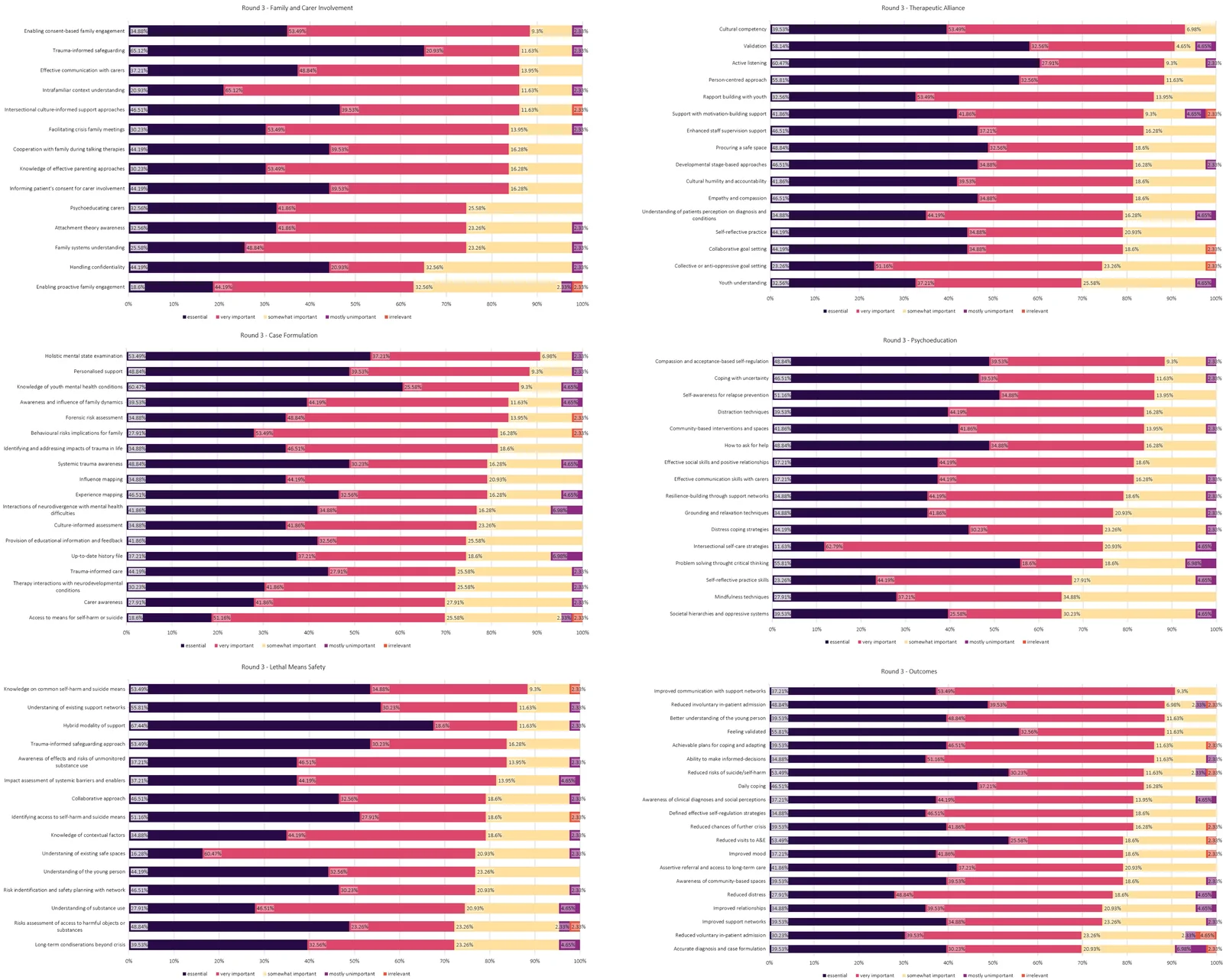
Results from round 3 of the Delphi exercise.

Most of the elements exceeded the ≥70% threshold for consensus. The results from round 3 are shown in figure 2 and in online supplemental table S5.

As shown in figure 2, following round 3, all but two elements in the domain of *Family and Carer Involvement* met the 70% consensus threshold. The highest agreement level was observed for *enabling consent-based family engagement* (88.4%), *effective communication with carers* (86%) and *intrafamilial context understanding* (86%). Only *proactive family engagement* (62.8%) and *handling confidentiality* (65.1%) fell below consensus. The *Therapeutic Alliance* elements showed the strongest overall consensus levels. Most elements exceeded 80% agreement, with *cultural competency* (93%), *validation* (90.7%) and *person-centred approach* (88.4%) among those seen as *essential/very important* with the strongest consensus.

Consensus was, again, achieved for nearly all elements in the domain of *Case Formulation*. Those with the highest levels of agreement included *holistic mental state examination* (90.7%), *personalised support* (88.4%) and *knowledge of youth mental health conditions* (86%). The elements of *access to means for self-harm or suicide* (69.8%) and *carer awareness* (69.8%) were just below the consensus threshold. For the *Psychoeducation* domain, participants reported high levels of agreement on the importance of skills promoting self-regulation and resilience; *compassion and acceptance-based self-regulation* (88.4*%), self-awareness for relapse prevention* (86%) and *coping with uncertainty* (86%) all showed strong consensus. Elements, such as *mindfulness techniques* (65.1%) and *societal hierarchies and oppressive systems* (65.1%) showed lower levels of agreement, narrowly failing to reach consensus.

High levels of consensus were also observed across the elements of *Lethal Means Safety*. In particular, participants reported strong agreement for *knowledge on common self-harm and suicide means* (88.4%), *trauma-informed safeguarding* (83.7%) and *understanding of existing support networks* (86%). Slightly lower levels of consensus were observed for *risk assessment of access to harmful objects/substances* (72.1%) and *long-term considerations beyond crisis* (72.1%).

Finally, *outcome*-related elements achieved consistently high levels of agreement, with *improved communication with support networks* (90.7%), *feeling validated* (88.4%), *better understanding of the young person* (88.4%) and *reduced involuntary inpatient admission* (88.4%) reaching the highest levels of consensus. Even those with the lowest levels of consensus (such as *accurate diagnosis and case formulation* (69.8%) and *reduced voluntary inpatient admission* (69.8%) very narrowly missed the 70% threshold.

The Theory of Change logic model developed from the findings is shown in online supplemental figure S2, along with some explanatory notes. Here, the ‘outcomes’ were further categorised into ‘activities’, ‘outputs’ (process outcomes) and ‘outcomes’ (impacts) in accordance with the Theory of Change.

## Discussion

This Delphi exercise established expert consensus, incorporating a broad range of stakeholders, on core clinical competencies and priority outcomes for CAMHS crisis services. High levels of agreement were observed, with over 90% of proposed elements meeting the consensus threshold. Overall, findings indicate that experts—including those with lived experience—prioritise relational, safety-oriented and personalised care over procedural, diagnostic or checklist-driven approaches.

Regarding clinical competencies, the highest levels of consensus were observed within the *Therapeutic Alliance* and *Lethal Means Safety* domains. *Cultural competency* (93%) and *validation* (90.7%) were rated as particularly essential, highlighting the perceived importance of rapport, cultural humility and validating a young person’s distress as foundations of effective crisis intervention. Within the *Case Formulation* domain, *personalised support* (88.4%) and a *holistic mental state examination* (90.7%) were prioritised, reflecting the value placed on understanding young people within their broader psychosocial and cultural contexts rather than relying solely on symptom-focused assessments.

For outcomes, those rated most important included *improved communication with support networks* (90.7%), *young people feeling validated* (88.4%) and *better understanding of the young person* (88.4%). In this respect, it is noteworthy that two of these elements reflect changes in professional systems rather than the young person themselves, though are clearly viewed as valuable by the Delphi panel members. *Reduced involuntary inpatient admission* (88.4%) was also strongly endorsed, reinforcing the central role of crisis services in supporting young people safely within their communities. In contrast, more clinical or procedural outcomes, such as *accurate diagnosis* (69.8%), narrowly failed to reach consensus. Notably, a substantial proportion of those expressing reservations were young people, suggesting that being understood may be more immediately meaningful than diagnostic labelling during crisis care.

These findings align with existing evidence emphasising relational factors as key mechanisms of effective crisis care for self-harm and suicidality in young people.^18^ However, this study extends the literature by identifying validation and cultural competency as distinct, essential skills rather than generic communication competencies. The prioritisation of consent-based family involvement (88.4%) echoes evidence that young people value support from trusted adults while retaining agency over how this occurs.^19^ The differentiation between consent-based and proactive family involvement reflects established principles regarding adolescent autonomy and capacity, highlighting the complexity of balancing safeguarding with respect for young people’s rights.

From a workforce perspective, strong consensus on the importance of enhanced supervision and staff support (83.7%) is consistent with evidence linking emotional labour in crisis settings to burnout and reduced retention.^24 25^ Stakeholders appear to recognise that clinicians require robust support systems to sustain empathic, validating care.

The prominence of relational competencies likely reflects the composition of the panel, which predominantly comprised young adults with relevant lived experience. This may have shifted the emphasis from clinical procedures towards experiential aspects of care, such as feeling safe, understood and respected. Notably, some psychotherapeutic techniques, including mindfulness (65.1%), did not reach consensus. Qualitative feedback suggested that generic skills can feel invalidating if poorly timed or applied without sufficient relational grounding, aligning with emerging concerns about the universal applicability of mindfulness-based approaches.^26 27^ Compassion and acceptance-based approaches were preferred, emphasising validation over symptom suppression.

Similarly, ambivalence around diagnostic precision may reflect mixed experiences of diagnostic labelling, which can be experienced as either validating or stigmatising.^28 29^ The panel’s preference for *dynamic formulation* suggests a prioritisation of narrative understanding (‘what has happened to you?’) over categorical classification (‘what is wrong with you?’). While cultural competency achieved strong consensus, more explicit acknowledgement of systemic oppression did not, potentially reflecting the panel’s limited ethnic diversity and highlighting a gap between endorsement of culturally informed care and engagement with structural determinants of mental health.

### Strengths and limitations

Strengths include the equal status afforded to lived experience experts, modest attrition across rounds (62% completion), and the high representation of young adults with recent crisis service experience. These features align with WHO guidance on democratising mental health research.^16^ The Delphi method also enabled refinement of terminology, such as reframing ‘*lethal means restriction’* as ‘*lethal means safety’*.

Limitations include the predominance of female and White participants, potentially limiting cultural generalisability. The exclusion of under-18s may mean findings differ from the perspectives of current adolescent service users. Additionally, clinician participation declined across rounds, meaning consensus was weighted towards service-user perspectives, which may pose challenges for implementation. In this regard, while we did not actively recruit participants holding purely managerial roles some of the senior clinicians involved would likely have been involved with service development, improvement and routine evaluation. Nevertheless, it would have been useful to have explicitly enquired about such roles and recruited those in management positions.

### Implications for policy, practice and further research

The findings informed the development of a prototype digital training package for CAMHS crisis teams and support policy goals to reduce inpatient admissions through effective community-based care. However, such reductions are contingent on a well-supported workforce. Training programmes should therefore prioritise relational and emotional competencies alongside robust supervision structures. Approaches informed by dialectical behaviour therapy (DBT) skills may offer a practical framework, even where full DBT implementation is not feasible.

Many of the outcomes (figure 2) can be evaluated in routine practice or research. Some of these would be best measured via patient-reported outcome measures (PROMs), such as mood or distress levels. Others, such as feeling validated, would be best captured and quantified via patient-reported experience measures (PREMs).^30^ Some of the outcomes are also factors relating to the clinician (eg, ‘better understanding of the young person’). Some of these elements are best conceptualised as either ‘activities’ or ‘process outcomes’ (outputs) which form part of the putative pathway from inputs to impact in the logic model (online supplemental figure S2). It was also notable that none of the outcomes identified and agreed on were related to workforce factors, such as improved staff well-being and retention. This was likely due to the focus of the exercise being on improving the care of young people presenting in crisis. Nevertheless, given the nature of the work in crisis teams, further research specifically relating the quality of training and support for clinicians to workforce outcomes would seem to be a priority.

For service design, protocols should emphasise collaborative safety planning and consent-based family engagement, with transparent communication where confidentiality must be breached. Evaluation frameworks should incorporate both PROMs and PREMs, with emerging analytic tools offering opportunities to efficiently process qualitative feedback.

Given the potential challenges of recruiting participants to randomised trials,^10^ future research could examine the effectiveness of CAMHS crisis teams using quasi-experimental designs, evaluate training interventions and explore the needs of more diverse populations. Longitudinal studies are needed to assess whether prioritising relational, community-based crisis care translates into sustained reductions in self-harm and hospital admission.

## Conclusion

This study establishes a clear, expert consensus that effective CAMHS crisis care is defined not by procedural compliance or diagnostic precision, but by the quality of the therapeutic alliance and a holistic understanding of the young person. The findings provide an impetus to shift from a paternalistic, risk-aversive model to one that is collaborative, restorative and deeply relational. By prioritising validation, cultural competency and consent-based family involvement, crisis services can better align with the actual needs of young people. The message from this Delphi study is clear: young people in crisis, as well as their relatives and carers, do not just want to be ‘assessed’ or ‘processed’; they want to be understood, validated and supported to find safety within their own communities. Implementing the relevant competencies into standardised training offers a pathway to averting hospital admissions and fostering genuine recovery.

## Supplementary material

10.1136/bmjment-2026-302719 online supplemental file 1

## References

[1] NHS Digital (2022). Mental Health of Children and Young People in England 2022 - wave 3 follow up to the 2017 survey.

[2] Lewis P Reaching the tipping point. Children and young people’s mental health. https://www%20nhsconfed%20org/publications/reaching-tipping-point.

[3] Burn A-M, Holland J, Roe J (2025). Impact of young people’s admissions to adult mental health wards in England: national qualitative study. BJPsych Open.

[4] Gill F, Butler S, Pistrang N (2016). The experience of adolescent inpatient care and the anticipated transition to the community: Young people’s perspectives. J Adolesc.

[5] Moses T (2011). Adolescents’ perspectives about brief psychiatric hospitalization: what is helpful and what is not?. Psychiatr Q.

[6] Carpenter RA, Falkenburg J, White TP (2013). Crisis teams: systematic review of their effectiveness in practice. Psychiatrist.

[7] Holgersen KH, Pedersen SA, Brattland H (2022). A scoping review of studies into crisis resolution teams in community mental health services. Nord J Psychiatry.

[8] NHS England (2019). NHS Mental Health Implementation Plan 2019/20 – 2023/24.

[9] Syed S, Pascual-Sanchez A, Adesiyan P (2026). Systematic Review and Meta-Analysis: Effectiveness of Intensive Community Care Services and Psychosocial Interventions for Adolescents With Severe Mental Health Problems. J Am Acad Child Adolesc Psychiatry.

[10] Ougrin D, Thaventhiran T, Wong BH-C (2026). Intensive community care services for adolescents with acute psychiatric emergencies: trial feasibility findings from the pilot phase of a multi-centre randomised controlled trial. BMC Psychiatry.

[11] Care Quality Commission (2018). Review of children and young people’s mental health services.

[12] The National Health Service (2019). The NHS Long Term Plan.

[13] NHS England (2024). Urgent and Emergency Mental Health Care for Children and Young People: National Implementation Guidance.

[14] Dalkey N, Helmer O (1963). An Experimental Application of the DELPHI Method to the Use of Experts. Manage Sci.

[15] Nasa P, Jain R, Juneja D (2021). Delphi methodology in healthcare research: How to decide its appropriateness. World J Methodol.

[16] WHO (2025). Guidance on mental health policy and strategic action plans: module 1: introduction, purpose and use of the guidance.

[17] Okoli C, Pawlowski SD (2004). The Delphi method as a research tool: an example, design considerations and applications. Inf Manag.

[18] Meza JI, Zullo L, Vargas SM (2023). Practitioner Review: Common elements in treatments for youth suicide attempts and self-harm - a practitioner review based on review of treatment elements associated with intervention benefits. J Child Psychol Psychiatry.

[19] Edwards D, Carrier J, Csontos J (2024). Review: Crisis responses for children and young people - a systematic review of effectiveness, experiences and service organisation (CAMH-Crisis). Child Adolesc Ment Health.

[20] Braun V, Clarke V (2019). Reflecting on reflexive thematic analysis. Qual Res Sport Exerc Health.

[21] De Silva MJ, Breuer E, Lee L (2014). Theory of Change: a theory-driven approach to enhance the Medical Research Council’s framework for complex interventions. Trials.

[22] Beiderbeck D, Frevel N, von der Gracht HA (2021). Preparing, conducting, and analyzing Delphi surveys: Cross-disciplinary practices, new directions, and advancements. MethodsX.

[23] Smith NJ, van der Walt S (2015). Viridis, Magma, Inferno, and Plasma colormaps. https://bids.github.io/colormap.

[24] Adams R, Ryan T, Wood E (2021). Understanding the factors that affect retention within the mental health nursing workforce: a systematic review and thematic synthesis. Int J Ment Health Nurs.

[25] Johnson J, Hall LH, Berzins K (2018). Mental healthcare staff well-being and burnout: A narrative review of trends, causes, implications, and recommendations for future interventions. Int J Ment Health Nurs.

[26] Kuyken W, Ball S, Crane C (2022). Effectiveness and cost-effectiveness of universal school-based mindfulness training compared with normal school provision in reducing risk of mental health problems and promoting well-being in adolescence: the MYRIAD cluster randomised controlled trial. Evid Based Ment Health.

[27] Farias M (2022). DEBATE: The inevitable decline of mindfulness. Child Adolesc Ment Health.

[28] Bjønness S, Grønnestad T, Storm M (2020). I’m not a diagnosis: Adolescents’ perspectives on user participation and shared decision-making in mental healthcare. Scand J Child Adolesc Psychiatr Psychol.

[29] Thomson L, Newman K, Ewart C (2025). Barriers and facilitators to using standardised diagnostic assessments in child and adolescent mental health services: a qualitative process evaluation of the STADIA trial. Eur Child Adolesc Psychiatry.

[30] Kjøllesdal MKR, Iversen HH, Ellingsen-Dalskau LH (2025). Concurrent use and association of patient-reported experience and outcome measures in psychiatric and substance use disorder care: a scoping review. Front Health Serv.

