# Supplementary material for: Building consensus on the core clinical competencies and outcomes related to effective child and adolescent mental health services (CAMHS) crisis team provision: findings from a Delphi study
Source: BMJ Ment Health. 2026 Aug 11;29(1):e302719. doi: 10.1136/bmjment-2026-302719 (PMC13475351; doi:10.1136/bmjment-2026-302719)
Supplement: Supplementary file 1 [file bmjment-29-1-s001.pdf]

Supplementary Material for: Building consensus on the core clinical competencies and outcomes related to effective Child and Adolescent Mental Health Services (CAMHS) crisis team provision: findings from a Delphi study

Authors:

Clau Nader

Lucy Tindall

Kapil Sayal

Josephine Holland

Catherine E Hewitt

Paul A Tiffin

Correspondence to:

paul.tiffin@york.ac.uk

Introduction

This *Supplementary Material* document contains additional results and details in relation to the Delphi exercise conducted and reported in the main paper as entitled above. Section 1 contains supplementary results, including those from the first and second stage of the Delphi exercise. We also include a Theory of Change logic model. This postulates how the core staff competencies lead to the desired key outcomes (impacts) via the relevant activities and outputs. Explanatory notes also accompany the logic model. Section two contains the list of the preliminary codes developed when performing a thematic analysis on the free-text responses to stage one of the Delphi exercise.

Section 1: Supplementary results

Table S1. List of initial, suggested items for each area for round one of the Delphi exercise.

Area	Item
**Family and carer involvement**	– Knowledge of how families and other 'systems' work<br>– Knowledge about how people attach and develop relationships with parents/care givers<br>– Knowledge of effective approaches to parenting<br>– Rapport building with families and/or carers<br>– Involving parents/carers as co-therapists<br>– Hosting effective family sessions young person is distressed<br>– All of the above<br>– Other
**Therapeutic alliance**	– Rapport building and therapeutic alliance with young people in crisis or distress<br>– Collaborative goal setting<br>– Cultural competence<br>– Cultural humility<br>– Guiding and feeding back to young people to find motivation and support in generating positive changes<br>– Exploration, validation, and normalisation of emotional responses<br>– All of the above<br>– Other
**Case formulation**	– Knowledge on different, common mental health difficulties that affect young people<br>– Understanding neurodevelopmental conditions and their interactions with mental health difficulties<br>– Understanding and responding to the impact of trauma on young people's lives<br>– Identifying family relationships and dynamics and helping young people´s network understand the influence of these in young people's experience<br>– Providing information and feedback to young people and their families to help them understand and nurture their mental health<br>– All of the above<br>– Other
**Psychoeducation**	– Teaching young people how to practice mindfulness techniques<br>– Helping young people develop relaxation techniques and feel calmer using grounding exercises<br>– Teaching young people how to think critically and solve problems effectively<br>– Teaching young people how to communicate and socially relate with other people positively<br>– Teaching young people how to identify, understand, accept, and manage their emotions and stress responses through healthy coping mechanisms<br>– Empowering young people by guiding them in comprehending the root of their behaviours, beliefs, and responses in order to develop self-compassion and self-regulation strategies<br>– All of the above<br>– Other

**Lethal means restriction**	– Having awareness of the effects and possible dangers of taking medication without proper guidance or other psychotropic substances<br>– Being knowledgeable about the existing risks and consequences of accessing potential harmful objects or substances<br>– Helping young people and families identify possible risks<br>– Working together with young people and their parents/carers to create a safety plan<br>– All of the above<br>– Other
**Outcomes**	– Reduced distress<br>– Receiving an accurate mental health diagnosis<br>– Being able to cope better with daily life<br>– Improved mood<br>– Reduced chances of further crises<br>– Reduced chances of having to go to A&E Hospital Dept<br>– Reduced chances of going into a mental health in-patient unit (as a voluntary patient)<br>– Reduced chances of going into a mental health in-patient unit (as an involuntary ['detained'] patient)<br>– Better family relationships<br>– All of the above<br>– Other

Table S2. Results of the first round of Delphi Study.

Item per area	N = 69	Participants n	Participants %	Missing
**Case Formulation**				
Knowledge on different, common MH* difficulties that affect YP**		45	65.22%	24
Understanding neurodevelopmental conditions and interactions with MH difficulties		43	62.32%	26
Understanding and responding to the impact of trauma on YP's lives		45	65.22%	24
Identifying family relationships and dynamics and helping YP's network understand the influence of these in YP's experience		43	62.32%	26
Providing information and feedback to YP and their families to help them understand and nurture their MH		46	66.67%	23
Other		11	15.94%	58
**Family and Carer Involvement**				
Knowledge of how families and other 'systems' work		49	71.01%	20
Knowledge about how people attach and develop relationships with parents/carers		43	62.32%	26
Knowledge of effective approaches to parenting		43	62.32%	26
Rapport building with families and/or carers		56	81.16%	13
Involving parents/carers as co-therapists		57	82.61%	12
Hosting effective family sessions when YP are distressed		48	69.57%	21
Other		11	15.94%	58
**Psychoeducation**				
Teaching YP how to practice mindfulness techniques		49	71.01%	20
Helping YP develop relaxation techniques and feel calmer using grounding exercises		55	79.71%	14
Teaching YP how to think critically and solve problems effectively		44	63.77%	25
Teaching YP how to communicate and socially relate with other people positively		47	68.12%	22
Teaching YP how to identify, understand, accept, and manage their emotions and stress responses through healthy coping mechanisms		58	84.06%	11
Empowering YP by guiding them in comprehending the root of their behaviours, beliefs, and responses to develop self-compassion and self-regulation strategies		47	68.12%	22
Other		10	14.49%	59
**Therapeutic Alliance**				
Rapport building and therapeutic alliance with YP in crisis or distress		49	71.01%	20
Collaborative goal setting		50	72.46%	19
Cultural competence		50	72.46%	19
Cultural humility		43	62.32%	26
Guiding and feedbacking YP to find motivation and support in generating positive changes		51	73.91%	18
Exploration, validation, and normalisation of emotional responses		59	85.51%	10
Other		8	11.59%	61

**Lethal Means Safety**			
Having awareness of the effects and possible dangers of taking medication without proper guidance or other psychotropic substances	55	79.71%	14
Being knowledgeable about the existing risks and consequences of accessing potential harmful objects or substances	59	85.51%	10
Helping YP and families identify possible risks	65	94.20%	4
Working together with YP and their parents/carers to create a safety plan	64	92.75%	5
Other	10	14.49%	59
**Outcomes**			
Reduced distress	48	69.57%	21
Receiving an accurate MH diagnosis	38	55.07%	31
Being able to cope better with daily life	45	65.22%	24
Improved mood	48	69.57%	21
Reduced chances of further crises	56	81.16%	13
Reduced chances of having to go to A&E Hospital Dept	37	53.62%	32
Reduced chances of going into a MH in-patient unit (as a voluntary patient)	39	56.52%	30
Reduced chances of going into a MH in-patient unit (as an involuntary ['detained'] patient)	38	55.07%	31
Better family relationships	46	66.67%	23
Other	8	11.59%	61

Note. *MH: mental health; **YP: young people. (N=69) Voting results for the first round of the Delphi study, indicating areas which participants consider that should be included in a digital training package for youth crisis support workers.

Table S3. Free text response per domain for round 1 of Delphi exercise.

Domain	Participant comment
**Case formulation**	*"risk... what that means to CYP /families, what the limits are to people's ability to cope with risky behaviour; hope and optimisim for the future, capacity of the system around the CYP to support that CYP through the difficult time; context and its impact on the presenting crisis"*
	"Including developmental/systemic trauma for the parents and carers. Within this context to be up-to-date with safeguarding training including parental mental health (e.g. PAMIC)."
	"They should consider the impact of the patients health on their level of functioning eg in a school setting or sociability. it should be considered if support could be offered in settings other than just the home, if it allows them to leave the house and provide a helpful distraction that is more pro active in improving their quality of life and could lead to an inpatient admission being less likely if support is given for them to improve their mental health in the community and allowing them to function better"
	"They also should know that CYP can consent to interventions without parental consent even if under 16 (Fraser guidelines/Gillick competencies)"
	"The impact of the school setting. Which social care services have been utilised by the family (non-statutory as well as statutory"
	"Whilst the aim of the crisis team is clearly to support the young person, it felt that there was insufficient focus and time on understanding and listening to the carer perspective, which can actually add a lot of value and isn't always available when just engaging with the young person."
	"ability to carry out MSE; ability to pull together multiple information from differing sources; access to means; intent; parental mental health; forensic risk and MH; poly substance misuse and safeguarding concerns. Cultural norms including traveller risks."
	"Understanding a persons background and environment while assessing mental states"
	"A strong knowledge of what is healthy or within the normal range of human behaviour and emotions. An understanding of how to interact with anxiety and anxious parents or referring professionals."
	"Also understanding how neurodevelopmental conditions may interact with types of therapy; CBT often isn't as useful if you're autistic, for example."
	"Consider how the young person is being systemically oppressed"
**Family and Carer Involvement**	*"Strategies to cope with strong emotions for both CYP and families, signposting for services for coping strategy enhancement, peer support avenues and how to help families access other forms of support including self-help...maybe make this box bigger!"*
	"Cultural Competency"
	"It's important that they have an understanding of confidentiality and where it is appropriate to use it in order to keep patients safe but also develop a relationship of trust. It's very important when working with patients at risk of self harm and suicide that they feel safe to talk about their feelings honestly and truthfully without fear of information being passed to family if they are not comfortable for this to happen for example if there is a strained family relationship.

"Understanding of trauma informed care and also that not all young people may want family involved so they should first discuss with CYP who they want to be involved in their care and who is their trusted adult and not just default to parents."

"Our experience of the CAMHS crisis team was that they didn't really bring any expertise that really helped. They suggested standard distraction techniques that we already knew of and had tried, but they tried to get [name removed] to work out what would help her, when actually she was so far in crisis, that she didn't really have the capacity to own and manage her feelings and actions. It would have been more helpful for the crisis team members to understand better the individuals situation and symptoms, so that any crisis management could be more bespoke and tailored to that individual, rather than being very generic and somewhat cliched."

"Teaching parents to make self-soothe boxes for their young person"

"Child development and how this creates different crisis at times; adolescence; bullying and impact; technology enabled distress e.g. online bullying, pro-ana websites; trauma including polyvagal theory and Sensory integration responses to managing distress; Knowing when you are going to make things worse; frequent users of crisis lines; how to recognise loneliness"

"often the young person may not wish to have their situation involved with theri parents and working out a way to bridge this gap if possible"

"Listening skills, high level of cognitive and emotional empathy skills, good personal emotional regulation, compassion."

*"Caveated with also the knowledge about when to *not* involve families if it would be unsafe for the person accessing crisis support, for example if it would out someone to an unsupportive family."*

"I believe the more knowledge, ideas and understanding about families/ carers/relationships that a mental health support provider has to draw from the better, as long as they are used flexibly and critically and not as ,"one size fits all". I believe that flexibility within a service is integral and services should be able to involve families in different ways depending on the needs and wants of the young person being supported. I believe all the ways suggested should be available when possible, but should be thought through and considered carefully alongside the young person being supported to ensure the experience is helpful and not harmful. I believe all young people should have the choice (and even encouraged) to have a separate mental health worker to the worker who is working with their caregivers or them and their families together. This is for reasons such as emotional safety and trust building."

Psychoeducation

"distraction, how to ask for help, how to understand the emotional build-up, places to get help, own triggers, relapse prevention strategies, self soothing strategies (pers, music, friends, tv, etc)"

"It is beneficial when Crisis team members are trained in DBT skills, particularly distress tolerance and emotion regulation. Parents should also be taught these skills so that they can support the young person and use apply them themselves in times of crisis."

"How to build resilience through support networks"

"elements of DBT, such as distress tolerance skills and emotional regulation skills. if there is no access to therapy or the patient is unable to access therapy due to their condition, individual skills might be more appropriate to access and crisis workers could

work with the patient to find skills that work and are effective for them whilst they are in crisis"

"As crisis teams for CYP rarely have an established relationship with the young person and often have limited contact they are unlikely to have the time to do most of these."

"Whilst all of the above options might help the young person, it is not really the role of a crisis service to educate the young person on these techniques. Once the young person is out of crisis, these techniques and guidance would be the role of generic CAMHS or another organisation. The role of crisis is to stop immediate physical or mental harm, not to provide longer term care. The role of the crisis team is to ensure the young person and their family/carer are signposted to these sources of support."

"All of these are useful if the young person is seen early enough by services, but in my experience, once the young person is in crisis, it is way too far down the line to think about mindfulness and other techniques. So really it's a matter of timing and not leaving the young person for so long that they get so far into crisis, that it is much harder to navigate away from an in patient stay."

"communication with parents. Mindfulness but over a longer period of time. Grounding and stabilisation over relaxation. Regulating skills (distress tolerance); how to ask for help; recognising triggers, recognising maintaining factors. Developing formulation."

"Developing emotional literacy, developing emotional regulation skills, teaching skills for managing anger and anxiety, helping young people understand simple life and social realities such as it is unrealistic to want to be liked by everyone, sometimes things don't go to plan, uncertainty is a normal human experience, anxiety can be helpful in many situations etc"

"Giving young people the option to be taught about harmful hierarchal societal systems, and how these impact their own life and wellness. This can give young people the opportunity to reflect on how their own wellness may have been personally impacted. To then empower young people by teaching them the therapeutic and healing qualities in nurturing community, affirmative, collective action and challenging harmful systems and exclusive ideologies."

Therapeutic Alliance

"emotional resilience; how supervision/consultation helps practice; appreciation of context and time available in the development of relationships; understanding the psychopathology and how CYP/families make sense of them"

"Good Supervision support/Reflective Practice"

"Crisis workers should learn to not use generic distress tolerance skills when they are known to not be useful/ applicable to the patient. Often crisis teams suggest having a bath or a cup of tea and this is seen as not 'caring' or not validating the extreme of how they are feeling. If a patient is at high risk of suicide or struggle with extreme anxiety and are therefore unable or not safe to try the activity it is unhelpful and can cause a breakdown of the relationship leading to potentially the patient refusing to co operate with the support offered and causing the crisis intervention to be less effective."

"the last one is what young people say is most important along with not having their feelings dismissed or downplayed. Also ensuring parents do not dominate goal setting as sometimes young people can feel their views are dismissed in favour of the parental views."

"Developmental perspective - how to engage different ages; how to engage with parents of differing ability; neurodivergent and LD understanding; understanding forensic risk."

"Open mindedness, self reflection skills, good cognitive and emotional empathy skills"

"I haven't ticked goal setting because in my experience in therapy, goal setting made me more anxious when I wasn't progressing as fast as the NHS system wanted me to"

"1. Understanding the role white supremacy, capitalism, individualism have on the wellness of young people within society 2. A note about goal setting- I believe goal setting can perpetuate the above (in 1.) as it can promote the idea that young people are more worthy if they are productive in a capitalist ideal. It can also be helpful for many young people who feel empowered by collaborative goal setting. It is important that goal setting is not imposed."

Lethal Means Safety

"Ensuring that all contexts are considered to mitigate risk where possible. For example locking away medication at Grandparents house. And ensuring that the safety plan has considered school/college and that they have a copy."

"Cultural Barriers and enablers/Racism"

"It is not always useful in the long term to lock away or hide potential risks in the home or environment as when the patient returns here or are later reintroduced to risks in the environment they are faced with the same risk and increased responsibility to keep themself safe with the dangerous items around. whilst maybe essential in the short term it should be explored if there are other strategies that can be put in place to minimise risk"

"seeing the young person face to face not just on a crisis line call. "

"These are all essentials but again, timing is crucial, as once a young person is deep in crisis, then it's abit late to be identifying risks and creating safety plans. In my experience, once in crisis, the young person can be very adept at finding and using risky behaviours as a reaction to their distress. "

"To increase knowlege around risks that are not talked about as much such as the use of aerosols for self-harm or suicide"

"Safeguarding, forensic risks, poly substance misuse; access to means inc firearms; technology assisted bullying and other means that reduce safety such as suicide websites."

"By understanding what the young person is going through and offering another type of escape than dangerous ones"

"Creating a support network, understanding their own personal relapse signature, understanding their own personal triggers, the importance of care giving as well as receiving"

"Systemic failings, such as checking basic needs are being met, that the young person has frequent optional access to a safe community environment and safe adults, in addition to their home environment or school environments (schools are often an emotionally unsafe environment for young people, prepping them to be in a constant cycle of productivity with little rest in dictatorial environments) "

Outcomes

"access to longer term care (outpatient or inpatient, as appropriate), ability of family/carer to cope with this and future crises "

"While an accurate mental health diagnosis is important. It is equally important to have a thorough and dynamic formulation to support the young person to understand the difficulties that they are experiencing. Effective crisis intervention can help with overall engagement with mental health services and progress towards recovery. "

"if the cyp is already on the waiting list for caMHs ensure that their file is upto date and that their case is upgraded from routine to urgent. Also reduce risk of suicide or self harm and if not already waiting for caMHs service ensure they are referred. Dont just tell them to take a hot bath and have a hot chocolate and relax! "

"Some of these should be part of the offering of support services much before a crisis team is required. An accurate diagnosis and early support and treatment for those symptoms should reduce the need for crisis intervention. Overall,any effective support that reduces the need to go to A&E or in patient units would be preferable to waiting until the young person is in crisis and then trying to manage what can feel quite unmanageable. "

"clearer formulation and understanding. A feeling of being understood. Increased connection with family or support. timely assessment and access to inpatient. Not always about reducing sometimes about escalating need. "

"Improved support networks, improved plans for coping and adaptability"

"Mental health inpatient units have their uses so I'd focus on voluntarily versus involuntarily. "

"An accurate diagnosis for young people who will benefit from this. Workers to support young people to make informed decisions around this as much as possible. Give information about social and medical models of mental health. Give information about which diagnoses have negative stigma and likely to impact their life negatively in the future and which diagnoses are more likely to lead to positive outcomes. Have these conversations in relation to aspects of identity such as gender identity and racial identity as people of different identities have different experiences in a health care/ work/ other settings."

Figure S1. Results of the second round of the Delphi exercise (N=46).

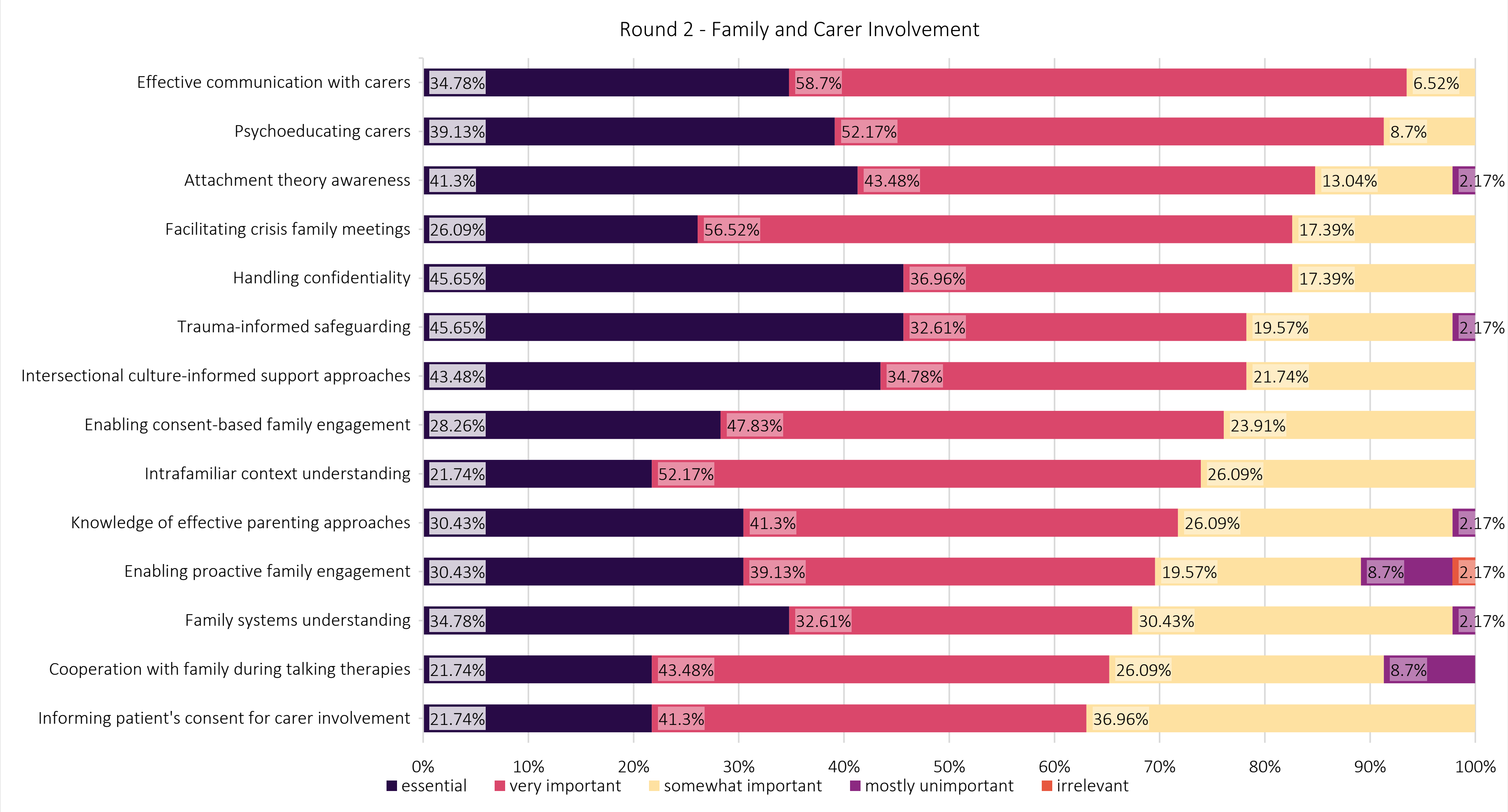

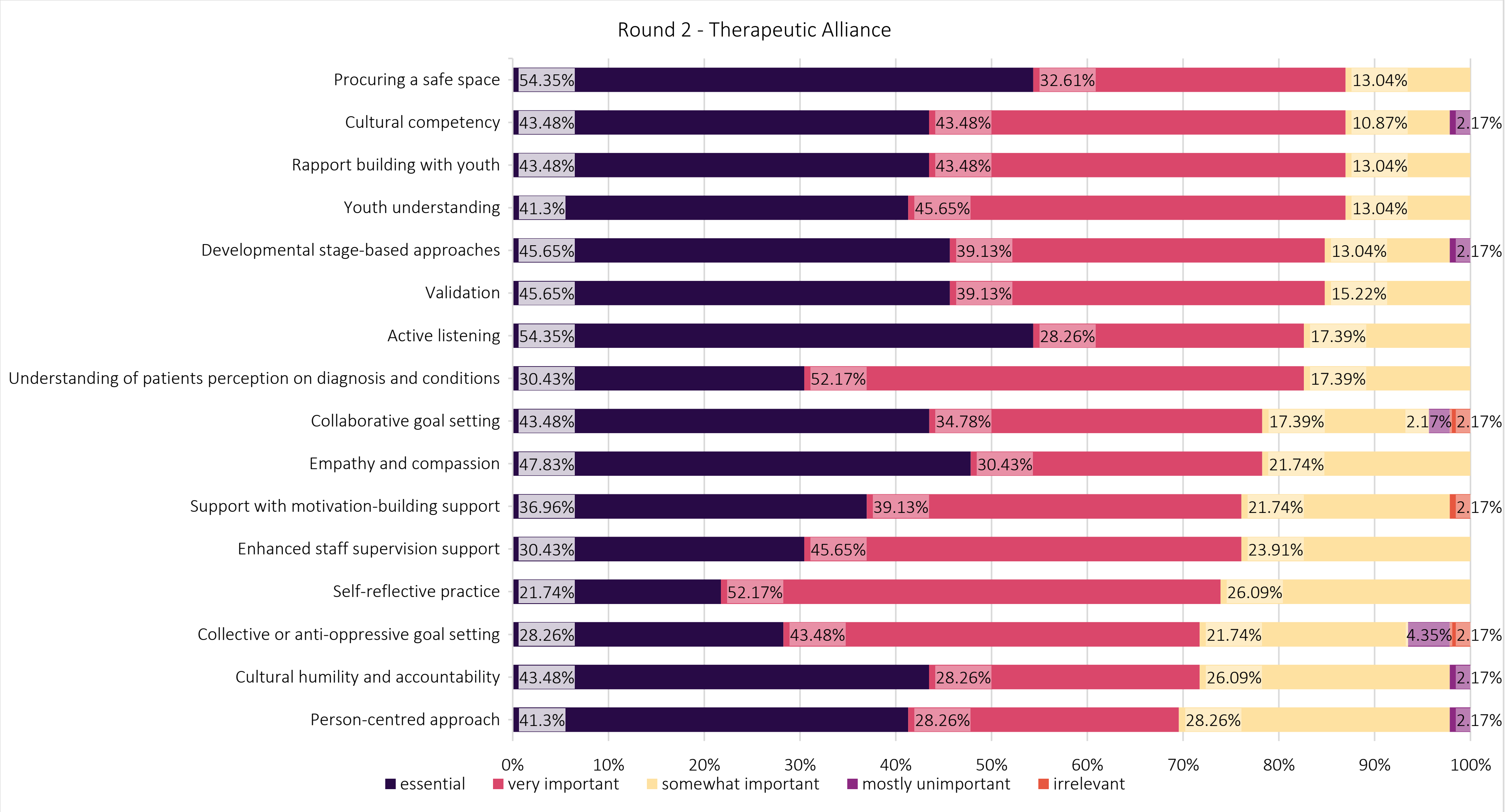

Round 2 - Therapeutic Alliance
Procuring a safe space
Cultural competency
Rapport building with youth
Youth understanding
Developmental stage-based approaches
Validation
Active listening
Understanding of patients perception on diagnosis and conditions
Collaborative goal setting
Empathy and compassion
Support with motivation-building support
Enhanced staff supervision support
Self-reflective practice
Collective or anti-oppressive goal setting
Cultural humility and accountability
Person-centred approach
54.35% 32.61% 13.04%
43.48% 43.48% 10.87% 2.17%
43.48% 43.48% 13.04%
41.3% 45.65% 13.04%
45.65% 39.13% 13.04% 2.17%
45.65% 39.13% 15.22%
54.35% 28.26% 17.39%
30.43% 52.17% 17.39%
43.48% 34.78% 17.39% 2.17% 2.17%
47.83% 30.43% 21.74%
36.96% 39.13% 21.74% 2.17%
30.43% 45.65% 23.91%
21.74% 52.17% 26.09%
28.26% 43.48% 21.74% 4.35% 2.17%
43.48% 28.26% 26.09% 2.17%
41.3% 28.26% 28.26% 2.17%
0% 10% 20% 30% 40% 50% 60% 70% 80% 90% 100%
essential
very important
somewhat important
mostly unimportant
irrelevant

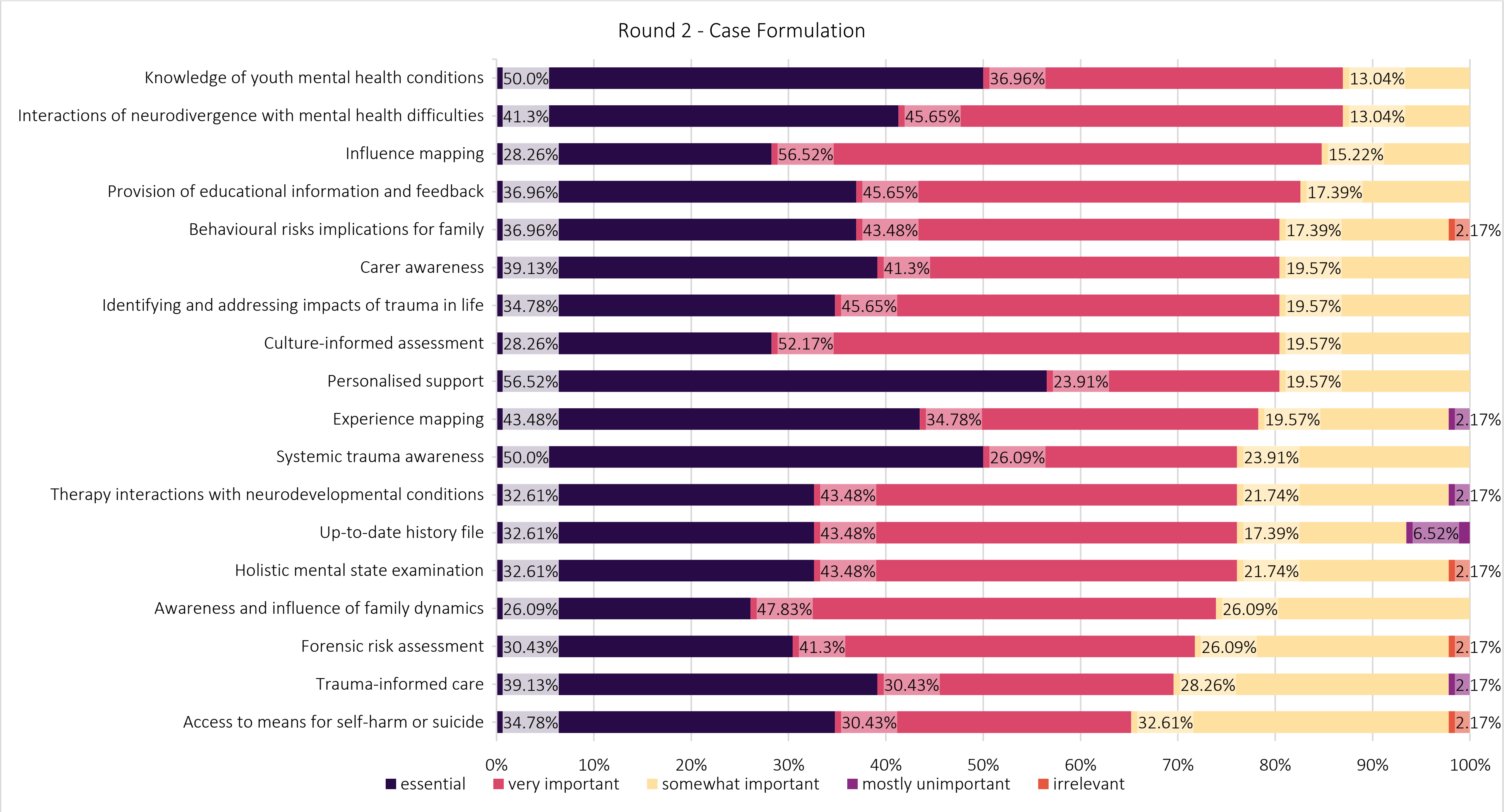
Round 2 - Case Formulation
Knowledge of youth mental health conditions
50.0%
36.96%
13.04%
Interactions of neurodivergence with mental health difficulties
41.3%
45.65%
13.04%
Influence mapping
28.26%
56.52%
15.22%
Provision of educational information and feedback
36.96%
45.65%
17.39%
Behavioural risks implications for family
36.96%
43.48%
17.39%
2.17%
Carer awareness
39.13%
41.3%
19.57%
Identifying and addressing impacts of trauma in life
34.78%
45.65%
19.57%
Culture-informed assessment
28.26%
52.17%
19.57%
Personalised support
56.52%
23.91%
19.57%
Experience mapping
43.48%
34.78%
19.57%
2.17%
Systemic trauma awareness
50.0%
26.09%
23.91%
Therapy interactions with neurodevelopmental conditions
32.61%
43.48%
21.74%
2.17%
Up-to-date history file
32.61%
43.48%
17.39%
6.52%
Holistic mental state examination
32.61%
43.48%
21.74%
2.17%
Awareness and influence of family dynamics
26.09%
47.83%
26.09%
Forensic risk assessment
30.43%
41.3%
26.09%
2.17%
Trauma-informed care
39.13%
30.43%
28.26%
2.17%
Access to means for self-harm or suicide
34.78%
30.43%
32.61%
2.17%
0%
10%
20%
30%
40%
50%
60%
70%
80%
90%
100%
essential
very important
somewhat important
mostly unimportant
irrelevant

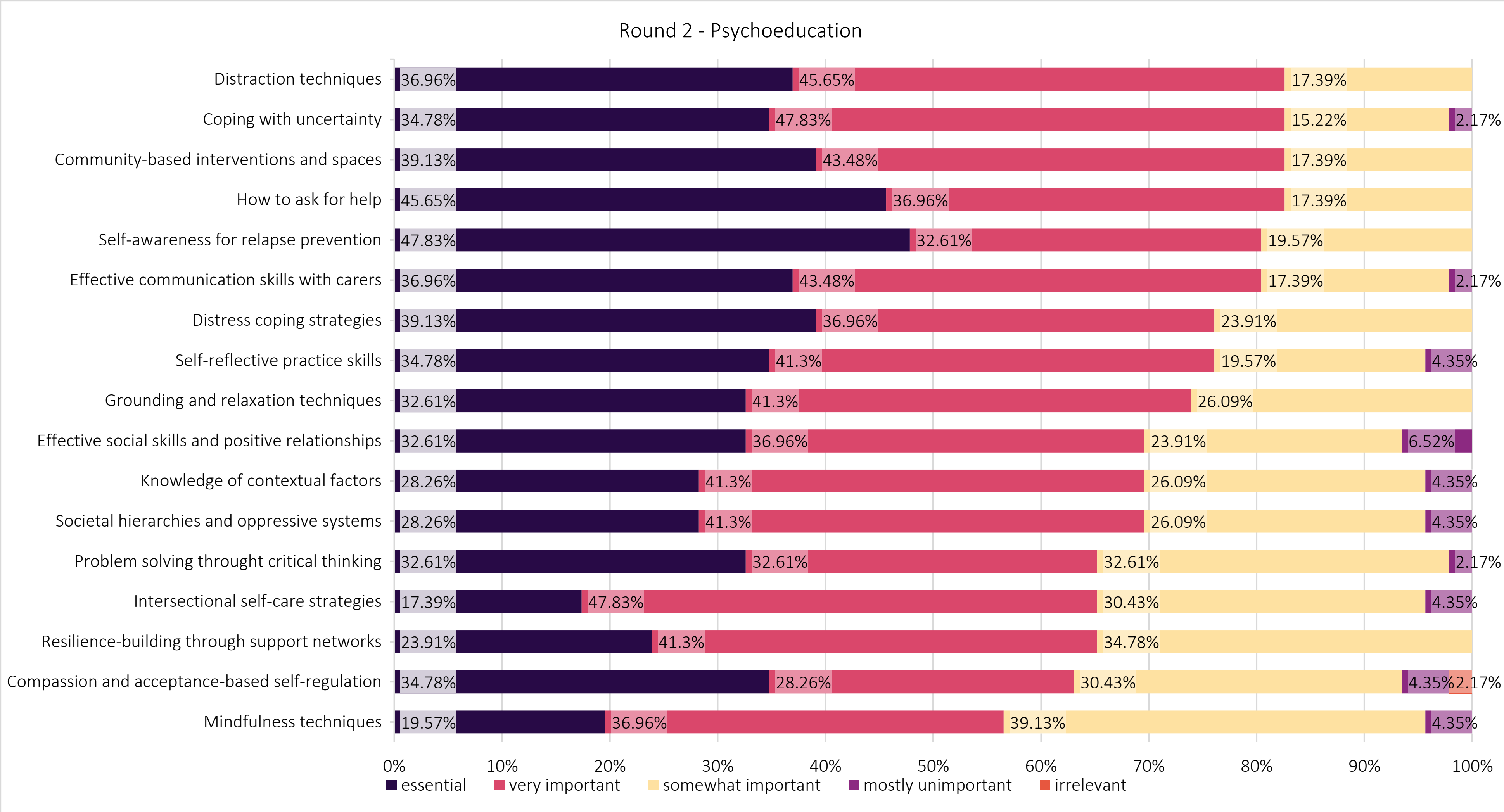

Round 2 - Psychoeducation
Distraction techniques
Coping with uncertainty
Community-based interventions and spaces
How to ask for help
Self-awareness for relapse prevention
Effective communication skills with carers
Distress coping strategies
Self-reflective practice skills
Grounding and relaxation techniques
Effective social skills and positive relationships
Knowledge of contextual factors
Societal hierarchies and oppressive systems
Problem solving throught critical thinking
Intersectional self-care strategies
Resilience-building through support networks
Compassion and acceptance-based self-regulation
Mindfulness techniques
36.96% 45.65% 17.39%
34.78% 47.83% 15.22% 2.17%
39.13% 43.48% 17.39%
45.65% 36.96% 17.39%
47.83% 32.61% 19.57%
36.96% 43.48% 17.39% 2.17%
39.13% 36.96% 23.91%
34.78% 41.3% 19.57% 4.35%
32.61% 41.3% 26.09%
32.61% 36.96% 23.91% 6.52%
28.26% 41.3% 26.09% 4.35%
28.26% 41.3% 26.09% 4.35%
32.61% 32.61% 32.61% 2.17%
17.39% 47.83% 30.43% 4.35%
23.91% 41.3% 34.78%
34.78% 28.26% 30.43% 4.35% 2.17%
19.57% 36.96% 39.13% 4.35%
0% 10% 20% 30% 40% 50% 60% 70% 80% 90% 100%
essential
very important
somewhat important
mostly unimportant
irrelevant

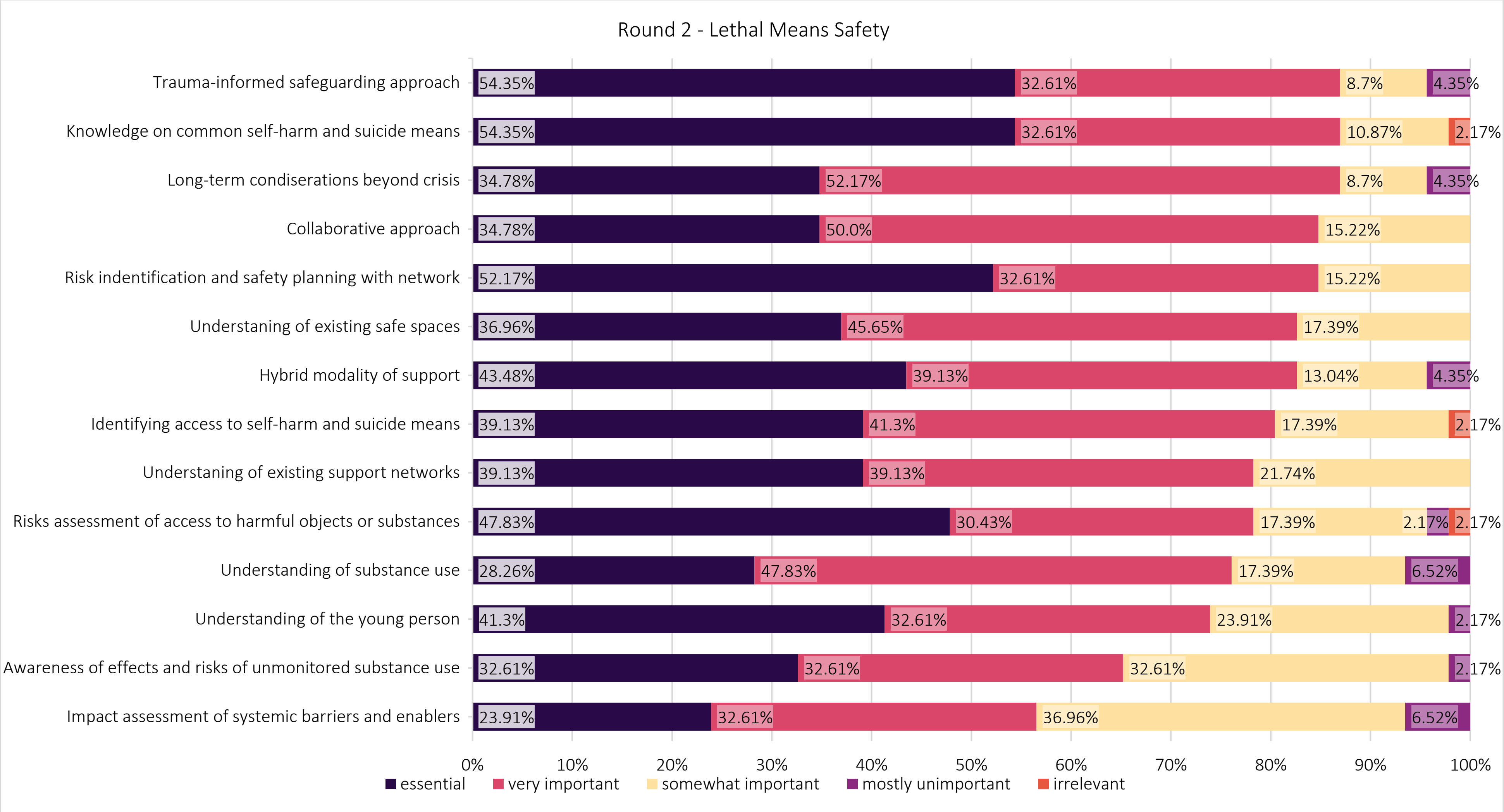
Round 2 - Lethal Means Safety
Trauma-informed safeguarding approach
Knowledge on common self-harm and suicide means
Long-term condiserations beyond crisis
Collaborative approach
Risk indentification and safety planning with network
Understaning of existing safe spaces
Hybrid modality of support
Identifying access to self-harm and suicide means
Understaning of existing support networks
Risks assessment of access to harmful objects or substances
Understanding of substance use
Understanding of the young person
Awareness of effects and risks of unmonitored substance use
Impact assessment of systemic barriers and enablers
54.35% 32.61% 8.7% 4.35%
54.35% 32.61% 10.87% 2.17%
34.78% 52.17% 8.7% 4.35%
34.78% 50.0% 15.22%
52.17% 32.61% 15.22%
36.96% 45.65% 17.39%
43.48% 39.13% 13.04% 4.35%
39.13% 41.3% 17.39% 2.17%
39.13% 39.13% 21.74%
47.83% 30.43% 17.39% 2.17% 2.17%
28.26% 47.83% 17.39% 6.52%
41.3% 32.61% 23.91% 2.17%
32.61% 32.61% 32.61% 2.17%
23.91% 32.61% 36.96% 6.52%
0% 10% 20% 30% 40% 50% 60% 70% 80% 90% 100%
essential
very important
somewhat important
mostly unimportant
irrelevant

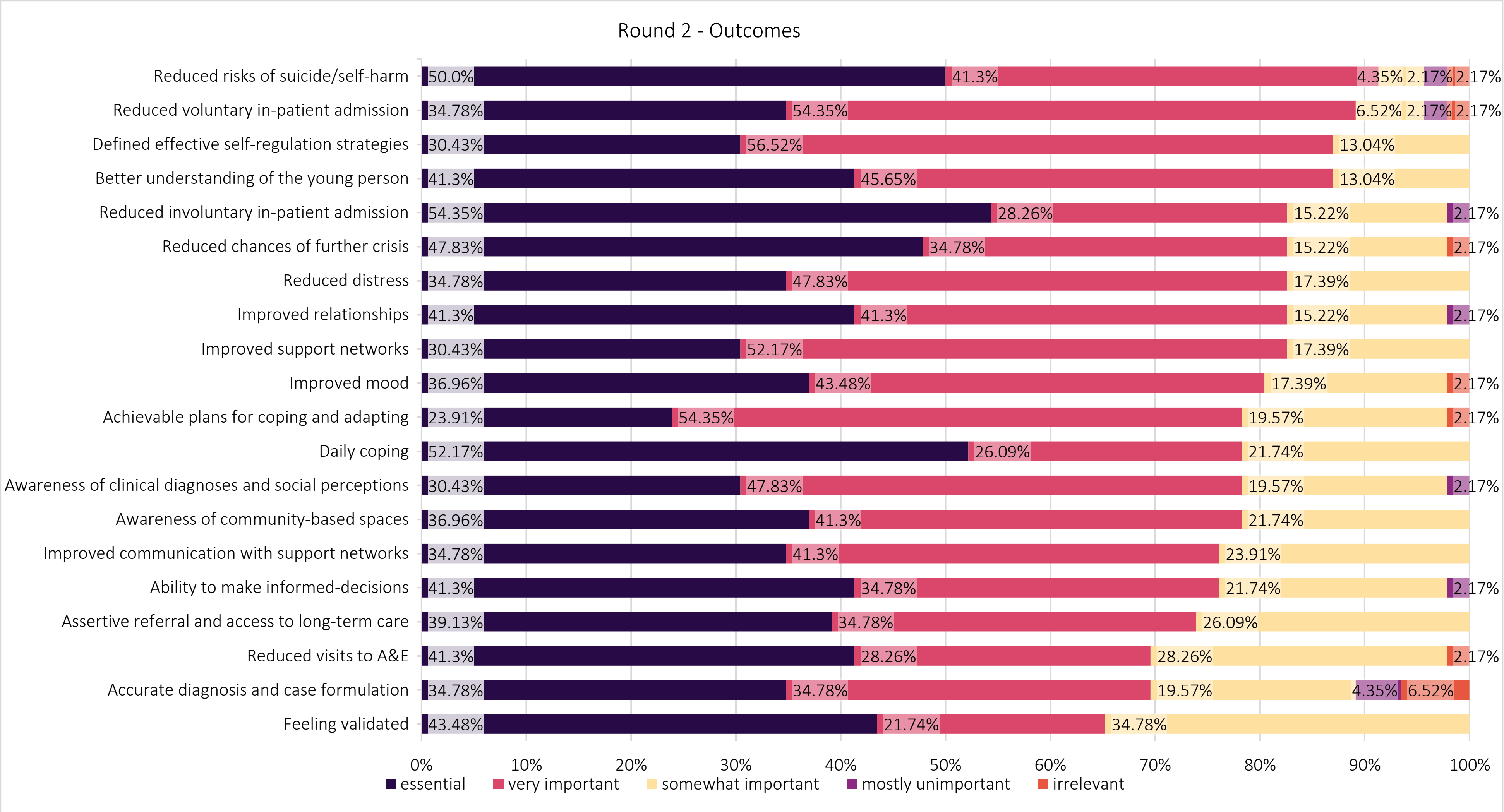
Round 2 - Outcomes
Reduced risks of suicide/self-harm 50.0% 41.3% 4.35% 2.17% 2.17%
Reduced voluntary in-patient admission 34.78% 54.35% 6.52% 2.17% 2.17%
Defined effective self-regulation strategies 30.43% 56.52% 13.04%
Better understanding of the young person 41.3% 45.65% 13.04%
Reduced involuntary in-patient admission 54.35% 28.26% 15.22% 2.17%
Reduced chances of further crisis 47.83% 34.78% 15.22% 2.17%
Reduced distress 34.78% 47.83% 17.39%
Improved relationships 41.3% 41.3% 15.22% 2.17%
Improved support networks 30.43% 52.17% 17.39%
Improved mood 36.96% 43.48% 17.39% 2.17%
Achievable plans for coping and adapting 23.91% 54.35% 19.57% 2.17%
Daily coping 52.17% 26.09% 21.74%
Awareness of clinical diagnoses and social perceptions 30.43% 47.83% 19.57% 2.17%
Awareness of community-based spaces 36.96% 41.3% 21.74%
Improved communication with support networks 34.78% 41.3% 23.91%
Ability to make informed-decisions 41.3% 34.78% 21.74% 2.17%
Assertive referral and access to long-term care 39.13% 34.78% 26.09%
Reduced visits to A&E 41.3% 28.26% 28.26% 2.17%
Accurate diagnosis and case formulation 34.78% 34.78% 19.57% 4.35% 6.52%
Feeling validated 43.48% 21.74% 34.78%
0% 10% 20% 30% 40% 50% 60% 70% 80% 90% 100%
essential
very important
somewhat important
mostly unimportant
irrelevant

Table S2. Results of round two of the Delphi exercise.

Survey item N = 46	Participants (n, %) consensus in		consensus out		no consensus	
**Family and carer involvement**						
Informing patient's consent for carer involvement	29	63.0%	0	0.0%	17	37.0%
Cooperation with family during talking therapies	30	65.2%	4	8.7%	12	26.1%
Family systems understanding	31	67.4%	1	2.2%	14	30.4%
Enabling proactive family engagement	32	69.6%	5	10.9%	9	19.6%
Knowledge of effective parenting approaches	33	71.7%	1	2.2%	12	26.1%
Intrafamilial context understanding	34	73.9%	0	0.0%	12	26.1%
Enabling consent-based family engagement	35	76.1%	0	0.0%	11	23.9%
Intersectional culture-informed support approaches	36	78.3%	0	0.0%	10	21.7%
Trauma-informed safeguarding	36	78.3%	1	2.2%	9	19.6%
Handling confidentiality	38	82.6%	0	0.0%	8	17.4%
Facilitating crisis family meetings	38	82.6%	0	0.0%	8	17.4%
Attachment theory awareness	39	84.8%	1	2.2%	6	13.0%
Psychoeducating carers	42	91.3%	0	0.0%	4	8.7%
Effective communication with carers	43	93.5%	0	0.0%	3	6.5%
**Therapeutic Alliance**						
Person-centred approach	32	69.6%	1	2.2%	13	28.3%
Cultural humility and accountability	33	71.7%	1	2.2%	12	26.1%
Collective or anti-oppressive goal setting	33	71.7%	3	6.5%	10	21.7%
Self-reflective practice	34	73.9%	0	0.0%	12	26.1%
Enhanced staff supervision support	35	76.1%	0	0.0%	11	23.9%
Support with motivation-building support	35	76.1%	1	2.2%	10	21.7%
Empathy and compassion	36	78.3%	0	0.0%	10	21.7%
Collaborative goal setting	36	78.3%	2	4.3%	8	17.4%
Active listening	38	82.6%	0	0.0%	8	17.4%
Understanding of patients' perception on diagnosis	38	82.6%	0	0.0%	8	17.4%
Validation	39	84.8%	0	0.0%	7	15.2%
Developmental stage-based approaches	39	84.8%	1	2.2%	6	13.0%
Rapport building with youth	40	87.0%	0	0.0%	6	13.0%
Procuring a safe space	40	87.0%	0	0.0%	6	13.0%
Cultural competency	40	87.0%	1	2.2%	5	10.9%
Youth understanding	40	87.0%	0	0.0%	6	13.0%
**Case formulation**						
Access to means for self-harm or suicide	30	65.2%	1	2.2%	15	32.6%
Trauma-informed care	32	69.6%	1	2.2%	13	28.3%
Forensic risk assessment	33	71.7%	1	2.2%	12	26.1%
Awareness and influence of family dynamics	34	73.9%	0	0.0%	12	26.1%
Holistic mental state examination	35	76.1%	1	2.2%	10	21.7%
Up-to-date history file	35	76.1%	3	6.5%	8	17.4%
Therapy interactions with neurodevelopmental conditions	35	76.1%	1	2.2%	10	21.7%

Systemic trauma awareness	35	76.1%	0	0.0%	11	23.9%
Experience mapping	36	78.3%	1	2.2%	9	19.6%
Personalised support	37	80.4%	0	0.0%	9	19.6%
Culture-informed assessment	37	80.4%	0	0.0%	9	19.6%
Identifying and addressing impacts of trauma in life	37	80.4%	0	0.0%	9	19.6%
Behavioural risks implications for family	37	80.4%	1	2.2%	8	17.4%
Carer awareness	37	80.4%	0	0.0%	9	19.6%
Provision of educational information and feedback	38	82.6%	0	0.0%	8	17.4%
Influence mapping	39	84.8%	0	0.0%	7	15.2%
Knowledge of youth mental health conditions	40	87.0%	0	0.0%	6	13.0%
Neurodivergence interactions w/ mental difficulties	40	87.0%	0	0.0%	6	13.0%
**Psychoeducation**						
Mindfulness techniques	26	56.5%	2	4.3%	18	39.1%
Compassion and acceptance-based self-regulation	29	63.0%	3	6.5%	14	30.4%
Intersectional self-care strategies	30	65.2%	2	4.3%	14	30.4%
Resilience-building through support networks	30	65.2%	0	0.0%	16	34.8%
Problem solving through critical thinking	30	65.2%	1	2.2%	15	32.6%
Effective social skills and positive relationships	32	69.6%	3	6.5%	11	23.9%
Societal hierarchies and oppressive systems	32	69.6%	2	4.3%	12	26.1%
Grounding and relaxation techniques	34	73.9%	0	0.0%	12	26.1%
Distress coping strategies	35	76.1%	0	0.0%	11	23.9%
Self-reflective practice skills	35	76.1%	2	4.3%	9	19.6%
Effective communication skills with carers	37	80.4%	1	2.2%	8	17.4%
Self-awareness for relapse prevention	37	80.4%	0	0.0%	9	19.6%
How to ask for help	38	82.6%	0	0.0%	8	17.4%
Community-based interventions and spaces	38	82.6%	0	0.0%	8	17.4%
Coping with uncertainty	38	82.6%	1	2.2%	7	15.2%
Distraction techniques	38	82.6%	0	0.0%	8	17.4%
**Lethal means safety**						
Impact assessment of systemic barriers and enablers	26	56.5%	3	6.5%	17	37.0%
Awareness of effects/risks of unmonitored substance use	30	65.2%	1	2.2%	15	32.6%
Knowledge of contextual factors	32	69.6%	2	4.3%	12	26.1%
Understanding of the young person	34	73.9%	1	2.2%	11	23.9%
Understanding of substance use	35	76.1%	3	6.5%	8	17.4%
Risks assessment of access to harmful objects/substances	36	78.3%	2	4.3%	8	17.4%
Understanding of existing support networks	36	78.3%	0	0.0%	10	21.7%
Identifying access to self-harm and suicide means	37	80.4%	1	2.2%	8	17.4%
Hybrid modality of support	38	82.6%	2	4.3%	6	13.0%
Understanding of existing safe spaces	38	82.6%	0	0.0%	8	17.4%
Risk identification and safety planning with network	39	84.8%	0	0.0%	7	15.2%
Collaborative approach	39	84.8%	0	0.0%	7	15.2%
Knowledge on common self-harm and suicide means	40	87.0%	1	2.2%	5	10.9%
Trauma-informed safeguarding approach	40	87.0%	2	4.3%	4	8.7%
Long-term considerations beyond crisis	40	87.0%	2	4.3%	4	8.7%

**Outcomes**						
Feeling validated	30	65.2%	0	0.0%	16	34.8%
Accurate diagnosis and case formulation	32	69.6%	5	10.9%	9	19.6%
Reduced visits to A&E	32	69.6%	1	2.2%	13	28.3%
Assertive referral and access to long-term care	34	73.9%	0	0.0%	12	26.1%
Ability to make informed-decisions	35	76.1%	1	2.2%	10	21.7%
Improved communication with support networks	35	76.1%	0	0.0%	11	23.9%
Daily coping	36	78.3%	0	0.0%	10	21.7%
Achievable plans for coping and adapting	36	78.3%	1	2.2%	9	19.6%
Awareness of community-based spaces	36	78.3%	0	0.0%	10	21.7%
Awareness of clinical diagnoses and social perceptions	36	78.3%	1	2.2%	9	19.6%
Improved mood	37	80.4%	1	2.2%	8	17.4%
Reduced distress	38	82.6%	0	0.0%	8	17.4%
Improved support networks	38	82.6%	0	0.0%	8	17.4%
Reduced chances of further crisis	38	82.6%	1	2.2%	7	15.2%
Reduced involuntary in-patient admission	38	82.6%	1	2.2%	7	15.2%
Improved relationships	38	82.6%	1	2.2%	7	15.2%
Better understanding of the young person	40	87.0%	0	0.0%	6	13.0%
Defined effective self-regulation strategies	40	87.0%	0	0.0%	6	13.0%
Reduced voluntary in-patient admission	41	89.1%	2	4.3%	3	6.5%
Reduced risks of suicide/self-harm	42	91.3%	2	4.3%	2	4.3%

Note. For each survey item, values from 5-point Likert-scale ratings ('irrelevant'= 1; 'mostly unimportant'= 2; 'somewhat important'= 3; 'very important'= 4; 'essential'= 5) were narrowed down into three categories to measure level of consensus: **consensus in** ('very important' and 'essential'), **consensus out** ('mostly unimportant' and irrelevant'), **no consensus** ('somewhat important').

Table S3. Results, in terms of consensus for round three of the Delphi exercise.

Survey item N = 43	consensus in		consensus out		no consensus	
	Participants (n, %)					
**Family and carer involvement**						
Enabling proactive family engagement	27	62.8%	2	4.7%	14	32.6%
Handling confidentiality	28	65.1%	1	2.3%	14	32.6%
Family systems understanding	32	74.4%	1	2.3%	10	23.3%
Attachment theory awareness	32	74.4%	1	2.3%	10	23.3%
Psychoeducating carers	32	74.4%	0	0.0%	11	25.6%
Informing patient's consent for carer involvement	36	83.7%	0	0.0%	7	16.3%
Knowledge of effective parenting approaches	36	83.7%	0	0.0%	7	16.3%
Cooperation with family during talking therapies	36	83.7%	0	0.0%	7	16.3%
Facilitating crisis family meetings	36	83.7%	1	2.3%	6	14.0%
Intrafamiliar context understanding	37	86.0%	1	2.3%	5	11.6%
Effective communication with carers	37	86.0%	0	0.0%	6	14.0%
Intersectional culture-informed support approaches	37	86.0%	1	2.3%	5	11.6%
Trauma-informed safeguarding	37	86.0%	1	2.3%	5	11.6%
Enabling consent-based family engagement	38	88.4%	1	2.3%	4	9.3%
**Therapeutic Alliance**						
Youth understanding	30	69.8%	2	4.7%	11	25.6%
Collective or anti-oppressive goal setting	32	74.4%	1	2.3%	10	23.3%
Collaborative goal setting	34	79.1%	1	2.3%	8	18.6%
Self-reflective practice	34	79.1%	0	0.0%	9	20.9%
Understanding of patients' perception on diagnosis	34	79.1%	2	4.7%	7	16.3%
Empathy and compassion	35	81.4%	0	0.0%	8	18.6%
Procuring a safe space	35	81.4%	0	0.0%	8	18.6%
Cultural humility and accountability	35	81.4%	0	0.0%	8	18.6%
Developmental stage-based approaches	35	81.4%	1	2.3%	7	16.3%
Enhanced staff supervision support	36	83.7%	0	0.0%	7	16.3%
Support with motivation-building support	36	83.7%	3	7.0%	4	9.3%
Rapport building with youth	37	86.0%	0	0.0%	6	14.0%
Person-centred approach	38	88.4%	0	0.0%	5	11.6%
Active listening	38	88.4%	1	2.3%	4	9.3%
Validation	39	90.7%	2	4.7%	2	4.7%
Cultural competency	40	93.0%	0	0.0%	3	7.0%
**Case formulation**						
Access to means for self-harm or suicide	30	69.8%	2	4.7%	11	25.6%
Carer awareness	30	69.8%	1	2.3%	12	27.9%
Trauma-informed care	31	72.1%	1	2.3%	11	25.6%
Therapy interactions w/ neurodevelopmental conditions	31	72.1%	1	2.3%	11	25.6%
Up-to-date history file	32	74.4%	3	7.0%	8	18.6%
Provision of educational information and feedback	32	74.4%	0	0.0%	11	25.6%
Culture-informed assessment	33	76.7%	0	0.0%	10	23.3%

Neurodivergence interactions w/ mental difficulties	33	76.7%	3	7.0%	7	16.3%
Systemic trauma awareness	34	79.1%	2	4.7%	7	16.3%
Experience mapping	34	79.1%	2	4.7%	7	16.3%
Influence mapping	34	79.1%	0	0.0%	9	20.9%
Identifying and addressing impacts of trauma in life	35	81.4%	0	0.0%	8	18.6%
Behavioural risks implications for family	35	81.4%	1	2.3%	7	16.3%
Forensic risk assessment	36	83.7%	1	2.3%	6	14.0%
Awareness and influence of family dynamics	36	83.7%	2	4.7%	5	11.6%
Knowledge of youth mental health conditions	37	86.0%	2	4.7%	4	9.3%
Personalised support	38	88.4%	1	2.3%	4	9.3%
Holistic mental state examination	39	90.7%	1	2.3%	3	7.0%
**Psychoeducation**						
Mindfulness techniques	28	65.1%	0	0.0%	15	34.9%
Societal hierarchies and oppressive systems	28	65.1%	2	4.7%	13	30.2%
Self-reflective practice skills	29	67.4%	2	4.7%	12	27.9%
Intersectional self-care strategies	32	74.4%	2	4.7%	9	20.9%
Distress coping strategies	32	74.4%	1	2.3%	10	23.3%
Problem solving through critical thinking	32	74.4%	3	7.0%	8	18.6%
Grounding and relaxation techniques	33	76.7%	1	2.3%	9	20.9%
Resilience-building through support networks	34	79.1%	1	2.3%	8	18.6%
Effective communication skills with carers	35	81.4%	1	2.3%	7	16.3%
Effective social skills and positive relationships	35	81.4%	0	0.0%	8	18.6%
How to ask for help	36	83.7%	0	0.0%	7	16.3%
Community-based interventions and spaces	36	83.7%	1	2.3%	6	14.0%
Distraction techniques	36	83.7%	0	0.0%	7	16.3%
Self-awareness for relapse prevention	37	86.0%	0	0.0%	6	14.0%
Coping with uncertainty	37	86.0%	1	2.3%	5	11.6%
Compassion and acceptance-based self-regulation	38	88.4%	1	2.3%	4	9.3%
**Lethal means safety**						
Risks assessment of access to harmful objects/substances	31	72.1%	2	4.7%	10	23.3%
Long-term considerations beyond crisis	31	72.1%	2	4.7%	10	23.3%
Understanding of substance use	32	74.4%	2	4.7%	9	20.9%
Understanding of the young person	33	76.7%	0	0.0%	10	23.3%
Understanding of existing safe spaces	33	76.7%	1	2.3%	9	20.9%
Risk identification and safety planning with network	33	76.7%	1	2.3%	9	20.9%
Knowledge of contextual factors	34	79.1%	1	2.3%	8	18.6%
Identifying access to self-harm and suicide means	34	79.1%	1	2.3%	8	18.6%
Collaborative approach	34	79.1%	1	2.3%	8	18.6%
Impact assessment of systemic barriers and enablers	35	81.4%	2	4.7%	6	14.0%
Awareness of effects/risks of unmonitored substance use	36	83.7%	1	2.3%	6	14.0%
Trauma-informed safeguarding approach	36	83.7%	0	0.0%	7	16.3%
Hybrid modality of support	37	86.0%	1	2.3%	5	11.6%
Understanding of existing support networks	37	86.0%	1	2.3%	5	11.6%
Knowledge on common self-harm and suicide means	38	88.4%	1	2.3%	4	9.3%

**Outcomes**						
Accurate diagnosis and case formulation	30	69.8%	4	9.3%	9	20.9%
Reduced voluntary in-patient admission	30	69.8%	3	7.0%	10	23.3%
Improved support networks	32	74.4%	1	2.3%	10	23.3%
Improved relationships	32	74.4%	2	4.7%	9	20.9%
Reduced distress	33	76.7%	2	4.7%	8	18.6%
Assertive referral and access to long-term care	34	79.1%	0	0.0%	9	20.9%
Improved mood	34	79.1%	1	2.3%	8	18.6%
Reduced visits to A&E	34	79.1%	1	2.3%	8	18.6%
Awareness of community-based spaces	34	79.1%	1	2.3%	8	18.6%
Reduced chances of further crisis	35	81.4%	1	2.3%	7	16.3%
Defined effective self-regulation strategies	35	81.4%	0	0.0%	8	18.6%
Awareness of clinical diagnoses and social perceptions	35	81.4%	2	4.7%	6	14.0%
Daily coping	36	83.7%	0	0.0%	7	16.3%
Reduced risks of suicide/self-harm	36	83.7%	2	4.7%	5	11.6%
Ability to make informed-decisions	37	86.0%	1	2.3%	5	11.6%
Achievable plans for coping and adapting	37	86.0%	1	2.3%	5	11.6%
Feeling validated	38	88.4%	0	0.0%	5	11.6%
Better understanding of the young person	38	88.4%	0	0.0%	5	11.6%
Reduced involuntary in-patient admission	38	88.4%	2	4.7%	3	7.0%
Improved communication with support networks	39	90.7%	0	0.0%	4	9.3%

Note. For each survey item, values from 5-point Likert-scale ratings ('Irrelevant'= 1; 'Mostly unimportant'= 2; 'Somewhat important'= 3; 'Very important'= 4; 'Essential'= 5) were narrowed down into three categories to measure level of consensus: consensus in ('Very important' and 'Essential'), consensus out ('Mostly unimportant' and 'Irrelevant'), no consensus ('Somewhat important').

Figure S2. A Theory of Change informed logic model postulating how the staff competencies lead to the desired key outcomes (impacts) via the relevant activities and outputs.

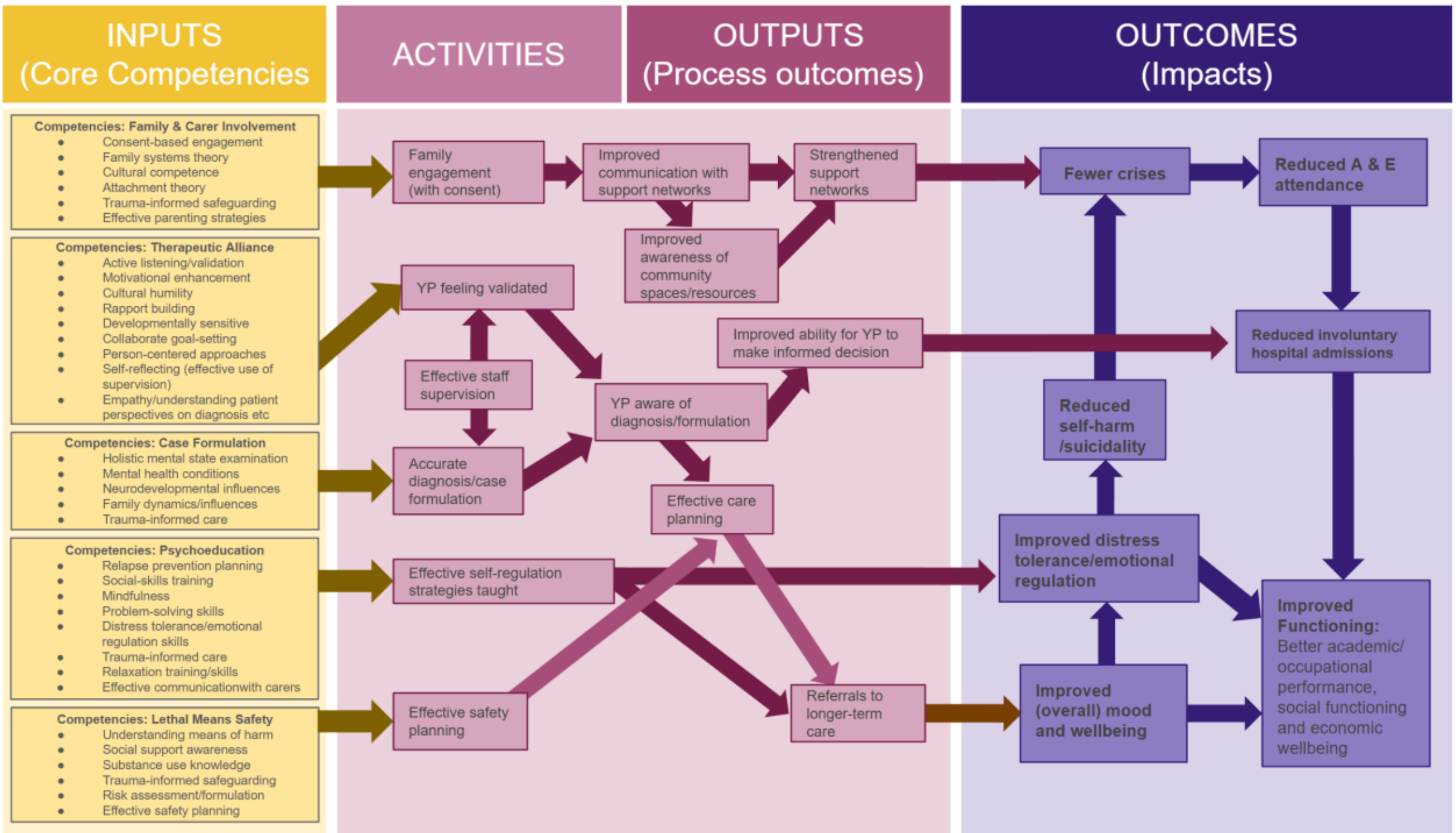

The Theory of Change informed logic model shown in Figure S2 illustrates how the key elements. Note, that, for visualisation purposes, some of the elements identified in the Delphi exercise have been abbreviated or otherwise summarised at times. The inputs here are the core competencies identified as desirable skills and knowledge in CAMHS crisis clinicians. The 'outcomes' identified as essential/important with good consensus also included 'activities' and 'outputs' (process outcomes) as well as actual impacts. These former two elements are thus conceptualised as mechanisms that mediate the relationship between the inputs (competencies) and the impacts. Most of the pathways should be self-explanatory. However, some specific, additional clarifications may be helpful. Firstly, effective staff supervision is an external factor, rather than a competency as such. However, training and support will be important in helping clinicians who are in supervisory roles themselves. Also, effective supervision is critical in psychologically containing staff working with young people and families experiencing string and challenging emotions. It is thus, important in reducing the risk of clinicians acting out any strong feelings themselves in ways which may make a young person feel invalidated or shamed. Hence, the pathway from "effective staff supervision" to "YP (Young Person) feeling validated". Effective supervision, both individual, and as a team, is also important in helping to develop accurate formulations and diagnoses. Moreover, young people who feel validated are more likely to come to a shared understanding with professionals, of the mental health and emotional challenges they face. A second feature worth noting is that this logic model is that only "involuntary ('formal') hospital admissions" are included in the impacts section. This is because there was substantially lower consensus regarding whether avoidance of voluntary inpatient admission was an important outcome (around 70% consensus) compared to involuntary admission (around 90% consensus). Moreover, in our previous public and patient involvement activities mental health service users have regularly expressed the view that consenting admissions to a period of hospital care, especially those that are planned, can be perceived as helpful.

Section 2: Preliminary coding for the free-text responses to stage one of the Delphi exercise

Case formulation

Code	References
ability to carry out MSE ~~(mental state examination)~~	1
ability to pull together multiple information from differing sources	1
~~knowledge if there is~~ access to means	1
an inpatient admission being less likely if support is given for them to improve their mental health in the community and allowing them to function better	1
be up-to-date with safeguarding training including parental mental health	1
capacity of the system	1
CBT often isn't as useful if you're autistic,	1
context and its impact on the presenting crisis	1
Cultural norms	1
CYP can consent to interventions without parental consent even if under 16	1
focus and time on understanding and listening to the carer perspective,	1
forensic risk and MH	1
hope and optimism for the future	1
how the young person is being systemically oppressed	1
if support could be offered in settings other than just the home, if it allows them to leave the house and provide a helpful distraction	1
impact of the patients health on their level of functioning eg in a school setting or sociability.	1
impact of the school setting	1
Implications of risky behaviour for family	1
Including developmental~systemic trauma for the parents and carers.	1
intent	1
parental mental health	1

poly substance misuse	1
safeguarding concerns	1
strong knowledge of what is healthy or within the normal range of human behaviours and emotions.	1
traveller risks	1
Understanding a persons background and environment	1
understanding how neurodevelopmental conditions may interact with types of therapy;	1
understanding of how to interact with anxiety and anxious parents or referring professionals.	1
what the limits are to people's ability to cope with risky behavior	1
Which social care services have been utilised by the family	1

Risk assessment and safety

Emphasis on mental state examination

Having an up-to-date history file

Awareness of access to means for self-harm or suicide

Understanding and assessment of forensic risks

Thorough training on safeguarding

Systemic trauma, social and environmental context

Awareness and understanding of how systemic trauma may influence and impact a young person's health (white supremacy, anti-Blackness, sexism, ableism, homophobia, transphobia)

Trauma-informed care for patients and their families/carers

Awareness of diverse cultural and religious norms

Identifying how a young person's background and environment influence or impact their health

Awareness of how a young person's health may impact their engagement in diverse settings (school, work) and its implications

Knowledge of alternative community-based interventions

Family and Carer Involvement

Improving listening to carers' perspective

Awareness of parental/carer's mental health and/or neurodevelopmental conditions

Considering the implications of risky behaviour for families

Considerations for accessibility

Understanding of how different therapy methods may interact with neurodevelopmental conditions (neurodivergence, learning difficulties)

Awareness of the capacity and limitations of the system in relation to a young person's needs

Ability to personalise and tailor support to the individual's needs rather than using generic, one-size-fits-all approaches

Understanding of different developmental stages of the young person

Developing therapeutic alliances	
**Code**	**References**
A note about goal setting- I believe goal setting can perpetuate the above (in 1.) as it can promote the idea that young people are more worthy if they are productive in a capitalist ideal.	1
appreciation of context and time available in the development of relationships	1
Crisis workers should learn to not use generic distress tolerance skills when they are known to not be useful~ applicable to the patient.	1
Developmental perspective	1
emotional resilience	1
ensuring parents do not dominate goal setting	1
goal setting made me more anxious when I wasn't progressing as fast as the NHS system wanted me to	1
good cognitive and emotional empathy skills	1
Good Supervision support~Reflective Practice	1
how supervision~consultation helps practice	1
how to engage different ages	1
how to engage with parents of differing ability	1
If a patient is at high risk of suicide or struggle with extreme anxiety and are therefore unable or not safe to try the activity it is unhelpful and can cause a breakdown of the relationship leading to potentially the patient refusing to co operate with them	1
It is important that goal setting is not imposed.	1
neurodivergent and LD understanding	1
Often crisis teams suggest having a bath or a cup of tea and this is seen as not 'caring' or not validating the extreme of how they are feeling.	1
Open mindedness	1
self reflection skills	1
sometimes young people can feel their views are dismissed in favour of the parental views.	1

it can also be helpful for many young people who feel empowered by collaborative goal setting.	1
understanding forensic risk	1
understanding the psychopathology and how CYP~families make sense of them	1
Understanding the role white supremacy, capitalism, individualism have on the wellness of young people within society	1
what young people say is most important along with not having their feelings dismissed or downplayed	1

Developing rapport

Empathy and compassion

Validation

Active listening skills

Person-centred support instead of "one size fits all" generic approached

Protected space for the young person
Collective goal setting (or goal setting with an anti-oppressive approach)

Enhanced supervision support

Self-reflective practice

Ability to engage with young people of different ages or at different developmental stages

Understanding of how young people and their families make sense of a condition/diagnosis

Involving family and carers as co-therapists	
**Code**	**References**
adolescence	1
all young people should have the choice (and even encouraged) to have a separate mental health worker to the worker who is working with their caregivers or them and their families together. This is for reasons such as emotional safety and trust building.	1
bullying and impact	1
Child development and how this creates different crisis at times	1
compassion	1
crisis management could be more bespoke and tailored to that individual,	1
Cultural Competency	1
develop a relationship of trust.	1
feel safe to talk about their feelings honestly and truthfully without fear of information being passed to family if they are not comfortable for this to happen f	1
flexibility within a service is integral	1
frequent users of crisis lines	1
good personal emotional regulation	1
high level of cognitive and emotional empathy skills	1
how to help families access other forms of support	1
how to recognise loneliness	1
if it would out someone to an unsupportive family.	1
if there is a strained family relationship.	1
Knowing when you are going to make things worse	1
knowledge about when to ~not~ involve families if it would be unsafe for the person accessing crisis support	1
Listening skills,	1
not all young people may want family involved	1

often the young person may not wish to have their situation involved with theri parents	1
peer support avenues	1
person may not wish to have their situation involved with theri parents and working out a way to bridge this gap if possible	1
rather than being very generic and somewhat cliched.	1
self-help	1
Sensory integration responses to managing distress	1
services should be able to involve families in different ways depending on the needs and wants of the young person being supported.	1
she didn't really have the capacity to own and manage her feelings and actions	1
should be thought through and considered carefully alongside the young person being supported to ensure the experience is helpful and not harmful.	1
should first discuss with CYP who they want to be involved in their care and who is their trusted adult and not just default to parents.	1
signposting for services for coping strategy enhahencement	1
Strategies to cope with strong emotions for both CYP and families	1
suggested standard distraction techniques that we already knew of and had tried,	1
Teaching parents to make self-soothe boxes for their young person	1
technology enabled distress e.g. online bullying, pro-ana websites	1
the CAMHS crisis team was that they didn't really bring any expertise that really helped.	1
the more knowledge, ideas and understanding about families~ carers~relationships that a mental health support provider has to draw from the better, used flexibly and critically and not as one size fits all	1
trauma including polyvagal theory	1
understand better the individuals situation and symptoms,	1
understanding of confidentiality and where it is appropriate to use it in order to keep patients safe	1
Understanding of trauma informed care	1

Confidentiality and trust

Maintaining appropriate confidentiality, especially in cases involving self-harm or strained family relationships

Supporting young people to give informed-consent to involve their parents or a nominated trusted adult in their care

Understanding the contextual relationship between the young person and their parents or carers

The challenge of involving family members in the care process when the young person may not want them involved

Not all young people may want their family involved

Ability to transfer knowledge on coping strategies to parents/carers

For example, creating self-soothing boxes

Ability to interact/communicate with parents/carers' with different mental health difficulties or disabilities

Cultural competency

Ability to offer support that is sensitive to the cultural backgrounds of young people and their family

Trauma-informed care

Training in safeguarding

Keeping young people safe whilst in risk

Code	References
schools are often an emotionally unsafe environment for young people,	1
access to means inc firearms	1
Creating a support network	1
Cultural Barriers and enablers~Racism	1
Ensuring that all contexts are considered to mitigate risk where possible	1
ensuring that the safety plan has considered school~college and that they have a copy.	1
forensic risks	1
increase knowlege around risks that are not talked about as much such as the use of aerosols for self-harm or suicide	1
It is not always useful in the long term to lock away or hide potential risks in the home or environment	1
it should be explored if there are other strategies that can be put in place to minimise risk	1
locking away medication at Grandparents house	1
once in crisis, the young person can be very adept at finding and using risky behaviors as a reaction to their distress.	1
other means that reduce safety such as suicide websites	1
poly substance misuse	1
prepping them to be in a constant cycle of productivity with little rest in dictatorial environments	1
Safeguarding	1
seeing the young person face to face not just on a crisis line call.	1
Systemic failings, such as checking basic needs are being met	1
technology assisted bullying	1
that the young person has frequent optional access to a safe community environment and safe adults, in addition to their home environment or school environments	1
the importance of care giving as well as receiving	1

timing is crucial, as once a young person is deep in crisis, then it's a late to be identifying risks and creating safety plans.	1
understanding their own personal relapse signature	1
understanding their own personal triggers	1
understanding what the young person is going through and offering another type of escape than dangerous ones	1
when the patient returns here or are later reintroduced to risks in the environment they are faced with the same risk and increased responsibility to keep themself safe with the dangerous items around.	

Identifying contextual factors (cultural, social, environmental, etc)

Cultural barriers and enablers (racism, homophobia, transphobia, sexism, ableism)

Identifying alternative environments where the young person is/feels safe

Identifying support networks in and out of school and home

Assessment of potential impacts of systemic failures in relation to intersectional social determinants

Understanding the young person's own self-awareness

In-person, face-to-face alternatives to online or phone line support

Long-term considerations beyond crisis

Knowledge on self-harm and suicide means, including technology-assisted and online platforms

Identifying any access to or usage of harmful means (firearms, suicide websites, aerosols)

Knowledge and understanding of polysubstance use, misuse, and dependence

Safeguarding

Outcomes of effective crisis support service	
**Code**	**References**
A feeling of being understood	1
ability of family~carer to cope with this and future crises	1
access to longer term care (outpatient or inpatient, as appropriate)	1
Also reduce risk of suicide or self harm and if not already waiting for camhs service ensure they are referred.	1
An accurate diagnosis and early support and treatment for those symptoms should reduce the need for crisis intervention.	1
An accurate diagnosis for young people who will benefit from this.	1
an accurate mental health diagnosis is important	1
clearer formulation and understanding	1
Dont just tell them to take a hot bath and have a hot chocolate and relax!	1
Effective crisis intervention can help with overall engagement with mental health services and progress towards recovery.	1
Give information about social and medical models of mental health	1
Give information about which diagnoses have negative stigma and likely to impact their life negatively in the future and which diagnoses are more likely to lead to positive outcomes. Have these conversations in relation to aspects of identity such as gender identity and racial identity as people of different identities have different experiences in a health care~ work~ other settings.	1
if the cyp is already on the waiting listfor camhs ensure that their file is upto date and that their case is upgraded from routine to urgent.	1
important to have a thorough and dynamic formulation to support the young person to understand the difficulties that they are experiencing	1
improved plans for coping and adaptability	1
Improved support networks	1
Increased connection with family or support	1
Mental health inpatient units have their uses so I'd focus on voluntarily versus involuntarily.	1

Not always about reducing sometimes about escalating need.	1
Overall,any effective support that reduces the need to go to A&E or in patient units would be preferable to waiting until the young person is in crisis and then trying to manage what can feel quite unmanageable.	1
Some of these should be part of the offering of support services much before a crisis team is required.	1
support the young person to understand the difficulties that they are experiencing	1
timely assessment and access to inpatient	1
Workers to support young people to make informed decisions around this as much as possible	1

Young people feeling understood, seen, validated

Ability to make informed-decisions

Accurate diagnosis and clear formulation

Referral and access to long-term care

Better understanding of the young person's experience and needs (by themselves and their network)

Understanding of different grounding and self-regulation techniques preferred by the young person

Awareness of diverse support alternatives that are available (at school, charities, peer support groups, etc)

Knowledge of different potential diagnoses as well as their societal and medical implications

Improved support networks

Increased connection with family or support

Reduced risks of suicide/self-harm

improved plans for coping and adaptability

Skills and training knowledge transfer

Code	References
All of these are useful if the young person is seen early enough by services,	1
anxiety can be helpful in many situations	1
beneficial when Crisis team members are trained in DBT skills	1
challenging harmful systems and exclusive ideologies.	1
communication with parents.	1
crisis teams for CYP rarely have an established relationship with the young person and often have limited contact they are unlikely to have the time to do most of these.	1
Developing emotional literacy,	1
Developing formulation.	1
distraction	1
distress tolerance	1
distress tolerance skills	1
elements of DBT	1
emotion regulation	1
emotional regulation skills	1
empower young people by teaching them the therapeutic and healing qualities in nurturing community, affirmative, collective action	1
developing emotional regulation skills	1
give young people the opportunity to reflect on how their own wellness may have been personally impacted	1
Giving young people the option to be taught about harmful hierarchal societal systems	1
Grounding and stabilisation over relaxation	1
harmful hierarchal societal systems, and how these impact their own life and wellness	1
helping young people understand simple life and social realities such as it is unrealistic to want to be liked by everyone	1
how to ask for help	2

How to build resilience through support networks	1
how to understand the emotional build-up	1
if there is no access to therapy or the patient is unable to access therapy due to their condition, individual skills might be more appropriate to access and crisis workers could work with the patient to find skills that work and are effective for them whi	1
it is not really the role of a crisis service to educate the young person on these techniques	1
matter of timing and not leaving the young person for so long that they get so far into crisis, that it is much harder to navigate away from an in patient stay.	1
Mindfulness but over a longer period of time	1
once the young person is in crisis, it is way too far down the line to think about mindfulness and other techniques.	1
Once the young person is out of crisis, these techniques and guidance would be the role of generic CAMHS or another organisation.	1
own triggers	1
Parents should also be taught these skills so that they can support the young person and use apply them themselves in times of crisis.	1
places to get help	1
recognising maintaining factors.	1
recognising triggers	1
Regulating skills (distress tolerance)	1
relapse prevention strategies	1
role of crisis is to stop immediate physical or mental harm, not to provide longer term care.	1
role of the crisis team is to ensure the young person and their family~carer are signposted to these sources of support.	1
self soothing strategies (peers, music, friends, tv, etc)	1
sometimes things don't go to plan	1
teaching skills for managing anger and anxiety	1
uncertainty is a normal human experience	1

Distraction techniques

Resilience building through support networks

Knowledge of community and collective healing spaces

Understanding emotions

Managing distress

Emotional regulation

Anger and anxiety management

Coping with uncertainty

Specific therapeutic strategies like DBT

Communication with parents and carers

Where and how to ask for help (signposting)

Relapse prevention strategies

Reflective practice and developing self-awareness (recognising own triggers, build-up of emotions, self-compassion)

Awareness of societal hierarchical systems and how these can impact a specific person
